# The impact of bilateral brachial-ankle pulse wave velocity difference on cardiovascular disease and all-cause mortality

**DOI:** 10.3389/fcvm.2023.1234325

**Published:** 2023-10-06

**Authors:** Mengyi Zheng, Xinyuan Zhang, Quanhui Zhao, Shuohua Chen, Xinying Guo, Chi Wang, Jost B. Jonas, Shouling Wu, Caixia Guo

**Affiliations:** ^1^ Cardiovascular Center, Beijing Tongren Hospital, Capital Medical University, Beijing, China; ^2^ Channing Division of Network Medicine, Brigham and Women’s Hospital and Harvard Medical School, Boston, MA, United States; ^3^ Department of Cardiology, Kailuan General Hospital, Tangshan, China; ^4^ Department of Cardiology, the Sixth Medical Center, Chinese PLA General Hospital, Beijing, China; ^5^ Department of Ophthalmology, Medical Faculty Mannheim, Heidelberg University, Mannheim, Germany

**Keywords:** arterial stiffness, bilateral difference, cardiovascular disease, all-cause mortality, clinical indicator

## Abstract

**Background:**

This study aims to investigate the association between an elevated bilateral pulse wave velocity difference (BPWVD) and cardiovascular diseases (CVDs) and all-cause mortality.

**Methods:**

This study included a total of 38,356 participants. A multivariable Cox proportional hazards regression was used to assess the association between high BPWVD and the increased risk of CVDs and all-cause mortality by calculating hazard ratios (HRs) with 95% confidence intervals.

**Results:**

A total of 1,213 cases of CVDs were identified over a mean duration of 6.19 years, including 886 cases of cerebral infarction (CI), 105 cases of intracerebral hemorrhage (ICH), and 222 cases of myocardial infarction (MI), along with 1,182 cases of all-cause mortality. The median BPWVD was 42 cm/s (19–80 cm/s). After adjusting for all confounders and baseline brachial-ankle PWV (baPWV), our analysis revealed a significant correlation between a higher risk of CVDs, MI, and all-cause mortality with an increase in BPWVD per standard deviation. HRs (95% confidence interval) were found to be 1.06 (1.01–1.11), 1.11 (1.02–1.21), and 1.07 (1.04–1.10), respectively. Among the participants with higher baPWV on the left side, the HRs (95% confidence interval) were 1.08 (1.02–1.14) for CVDs, 1.27 (1.10–1.46) for incident ICH, 1.16 (1.00–1.24) for incident MI, and 1.10 (1.07–1.15) for all-cause mortality, for per standard deviation increase in BPWVD.

**Conclusions:**

Our findings reveal a significant correlation between elevated BPWVD and the risks of developing CVDs and all-cause mortality. This highlights the importance of thoroughly evaluating BPWVD as a means of detecting individuals at risk for CVDs and mortality.

## Introduction

Arterial stiffness and atherosclerosis are independent risk factors for cardiovascular diseases (CVDs) and all-cause mortality (1). The brachial-ankle pulse wave velocity (baPWV) is a commonly employed non-invasive method in Asian countries for assessing arterial stiffness (2), and it has a strong correlation with carotid-femoral PWV (3). During the assessment of arterial stiffness, it is possible to measure blood pressure on all four extremities, as well as obtain the ankle-brachial index (ABI) and baPWV on the right and left side simultaneously (4). In this study, we observed inconsistencies in the bilateral brachial-ankle pulse wave velocity, which we referred to as the bilateral brachial-ankle pulse wave velocity difference (BPWVD).

Previous studies also found that the percent in the baPWV difference between the two sides was 4 ± 5% in subjects without any atherosclerotic factors, and it seems to relate to the delay in the calculated baPWV caused by arterial stenosis (5). The ABI and inter-arm difference (IAD) in blood pressure are the simple procedures used to diagnose peripheral artery disease (PAD) (6). In addition, high IAD ( ≥ 15 mmHg) and low ABI (ABI ≤ 0.9) (7, 8) have been consistently shown as independent predictors of atherosclerotic cardiovascular diseases (ASCVDs) and mortality. In individuals with severe atherosclerosis, such as PAD, the narrowing of the arteries due to stenosis affects the blood pressure between the two sides, resulting in a decrease in the accuracy of baPWV measurement (4, 9). Consequently, this leads to a greater discrepancy in baPWV values (abnormal difference) between left and right sides. It remains unclear whether an elevated BPWVD, similar to elevated IAD, is associated with a higher risk of CVD and all-cause mortality. Therefore, in order to clarify the clinical significance of BPWVD, we investigated the association between BPWVD and incident cardiovascular disease and all-cause mortality in the Kailuan cohort.

## Materials and methods

### Data sources

The Kailuan Study is a cohort study that focuses on a functional community population (10, 11). The study started in 2006, with participants being subjected to follow-up assessments every 2 years. During the second follow-up period (2010–2011), certain participants were subjected to baPWV detection. The study at Kailuan General Hospital was granted approval by the ethics committee in accordance with the Helsinki Declaration. Between 2010 and 2017, a total of 42,227 participants underwent baPWV measurements. All participants signed a written informed consent to participate in the study. Individuals who had outlier values of the baPWV [±5 standard deviation (SD)] (*n* = 239), those with a history of CVDs (*n* = 821) and atrial fibrillation (*n* = 328), and those with incomplete information on physical measurements were excluded from the study. Finally, this study included a total of 38,356 participants (Supplementary Figure S1). We compared the baseline characteristics for the inclusion and exclusion populations (Supplementary Table S1) (Registration No.: ChiCTR-TNC-11001489).

### BaPWV measurement

The baPWV detection was conducted from 7:00 a.m. to 9:00 a.m., while ensuring that the temperature of the examination room was maintained between 22°C and 25°C. Each participant was assessed by trained professionals. The baPWV values were collected using the detection device [BP-203RPE III networked device produced by Omron Healthcare (China) Co., Ltd.], and the four-limb blood pressure and ABI values were recorded simultaneously. Prior to the measurement, the participants were instructed to refrain from smoking, rest for more than 5 min, and maintain a quiet and supine posture during the measurement. Two replicate measurements were taken for each participant, and the average of the two sets of data was used as the final result (Supplementary Figure S2) (12). The definition of baseline baPWV is the average value of baPWV on the left and right sides at the baseline level.

### Definition of BPWVD

BPWVD is calculated as the absolute difference between left- and right-side baPWVs (9). The participants with BPWVD values higher than the highest quartile value (80 cm/s) were categorized as the “high BPWVD group,” whereas those with BPWVD values lower than 80 cm/s were categorized as the “normal BPWV group.” The IAD and the inter-ankle systolic blood pressure difference (IAND) were defined as the absolute difference in systolic blood pressure between both arms and ankles, respectively.

### Assessment of other variables

Following fasting for 8–12 h, 5 ml of elbow venous blood was collected from each participant during the follow-up. All blood biochemical indicators were measured at the central laboratory of Kailuan General Hospital using a Hitachi 747 automatic analyzer operated by specialized laboratory physicians. Using the CKD-EPI formula, the estimated glomerular filtration rate (eGFR) was calculated (13).

Blood pressure was measured between 7:00 a.m. and 9 a.m. on the survey. To ensure accuracy, all participants were instructed to abstain from smoking or consuming tea or coffee for a minimum duration of 30 min prior to the measurement. Three measurements were performed, separated by 1–2 min intervals, and the mean value was calculated. The mean arterial pressure (MAP) was calculated using the formula: 1/3 × systolic blood pressure (SBP) + 2/3 × diastolic blood pressure (DBP). Trained nurses measured the height, weight, heart rate, and waist circumference. Using the formula weight in kilograms divided by the height in meters squared, the body mass index (BMI) was calculated.

For demographic information, medications, and living habits, please refer to the previously published research (11, 14). During the 2016–2017 follow-up, supplementary information on the dominant hand was also gathered.

### Assessment of outcomes

The starting point of the study is marked by the time of the first baPWV test for each participant. Every year, trained professionals review the hospitalization diagnoses and document the outcomes of the participants at Kailuan General Hospital, as well as its affiliated hospitals and the designated hospitals covered by the city's medical insurance. The outcomes during the follow-up are CVDs and all-cause mortality, which includes cerebral infarction (CI), intracerebral hemorrhage (ICH), and myocardial infarction (MI). For individuals with multiple events (≥2 times), the time and event of the first occurrence were used as the outcome, while for those without any event, the end of follow-up was defined as the last available follow-up time, which was 31 December 2020 (15, 16). The diagnoses of MI (17), CI, and ICH (18) were based on both the patient's clinical symptoms and laboratory examination results.

### Statistical analysis

The mean and SD were used to describe the baseline characteristics of normally distributed continuous variables, while the median and interquartile range were used for skewed distribution continuous variables. Categorical variables were represented by frequencies and percentages. One-way analysis of variance or chi-square test was employed to compare the differences in continuous and categorical variables among various groups. Multivariate logistic regression, specifically stepwise regression, was used to investigate the risk factors associated with BPWVD.

A multivariate Cox proportional hazards regression model was used to analyze whether high BPWVD was associated with an increased risk of cardiovascular disease and all-cause mortality. Hazard ratios (HRs) and *P*-values for risks per SD increment of BPWVD were also calculated. Since the BPWVD value was the absolute value of the difference between the left and right sides, considering higher baPWV values on the left or right side may affect the results. Therefore, we divided the population into two subgroups based on participants with higher left or right baPWV values and repeated the above analysis in each group. Model 1 was adjusted for possible covariates, including age, sex, MAP, heart rate, fasting blood glucose (FBG), BMI, low-density lipoprotein cholesterol (LDL-C), high-density lipoprotein cholesterol (HDL-C), log-transformed high-sensitivity C-reactive protein (hs-CRP), uric acid (UA), eGFR, smoking status, alcohol consumption, physical activity, and dominant hand. Model 2 was further adjusted for lipid-lowering drugs, antihypertensive drugs, and antihyperglycemic drugs. IAD, IAND, and baseline baPWV were further adjusted in Model 3 and Model 4.

In addition, a series of sensitivity analyses were conducted to assess the consistency of the findings. First, considering the potential impact attributed to gender, one of the unmodifiable factors, we conducted stratified analyses and repeated measures. Second, considering the potential bias caused by peripheral artery disease, we excluded participants with ABI ≤ 0.9 or IAD ≥ 15 mmHg or both of these conditions in subsequent sensitivity analyses.

To assess the reproducibility of baPWV and BPWVD, 77 subjects without CVD were recruited as the repeatability validation population. BaPWV was measured three times according to the standard procedure, and we applied intraclass correlation coefficients (ICCs) to evaluate the reproducibility of the measurement results. Rosner's standardized criteria (2000) were utilized to assess the ICCs (19). In this validation population, the ICC is 0.58 for three repeated measurements and 0.63 for the last two repeated measurements, which indicate favorable reproducibility (Supplementary Tables S2–S6).

Finally, we fitted the time-dependent receiver operator characteristic (ROC) curves (20) and calculated the area under the curve (AUC) over time at 2, 4, 6, 8, and 10 years. The predictive values of BPWVD, ABI, and IAD in relation to CVD and all-cause mortality were compared over the follow-up.

All analyses were performed using SAS 9.4 (SAS Institute, Cary, NC, USA) and R software (version 3.6.0; R Core Team). Statistical significance was defined as two-sided *P*-values less than 0.05.

## Results

### Baseline characteristics

**Figure 1:**
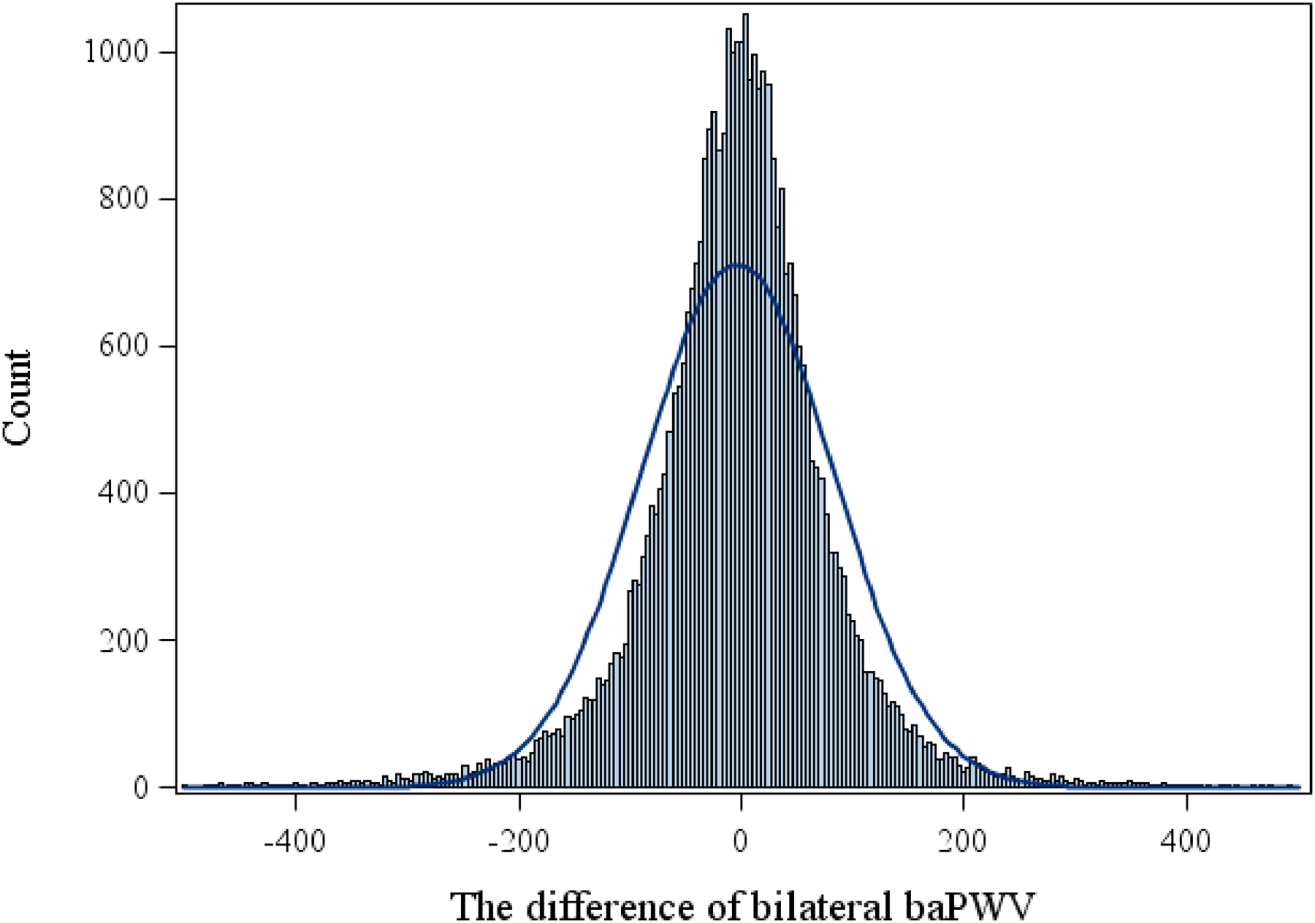
The histogram for difference of bilateral baPWV.

The mean age of the participants was 48.4 ± 12.7 years, with 27,691 (72.5%) being male. The baPWV measured on the right side was 1,477 ± 325 cm/s, whereas the baPWV measured on the left side was 1,482 ± 332 cm/s. The mean BPWVD was 62.9 ± 78.6 cm/s with a median of 42 cm/s and a third quartile value of 80 cm/s. The difference in bilateral baPWV was approximately normal, as shown in Figure 1. We therefore defined BPWVD ≥ 80 cm/s as the high BPWVD group, which consisted of 9,651 (25.2%) participants. The high BPWVD group was observed to have a significantly (*P* < 0.001) higher age, predominantly male, and a higher prevalence of cardiovascular risk factors in comparison with the normal BPWVD group (*P* < 0.001) (Table 1 and Supplementary Table S7).

**Table 1:** The baseline characteristics for the participants with high or normal bilateral baPWV difference.

	<80 cm/s (*n* = 28,705)	>80 cm/s (*n* = 9,651)	Total (*n* = 38,356)	*P*-value
Age (years)	47.1 ± 12.0	52.6 ± 14.0	48.4 ± 12.7	<0.001
Sex, *n* (%)	20,355 (71.2)	7,336 (76.3)	27,691 (72.5)	<0.001
Bilateral difference of baPWV (cm/s)	30.0 (14.0–49.0)	120 (95.0–166)	42.0 (19.0–80.0)	<0.001
RbaPWV (cm/s)	1,426 ± 280	1,630 ± 396	1,477 ± 325	<0.001
LbaPWV (cm/s)	1,426 ± 280	1,647 ± 410	1,482 ± 332	<0.001
Rabi	1.11 (1.05–1.17)	1.11 (1.04–1.18)	1.11 (1.05–1.18)	<0.001
Labi	1.11 (1.05–1.18)	1.11 (1.04–1.18)	1.11 (1.05–1.18)	<0.001
IAD (mmHg)	3.00 (1.00–6.00)	4.00 (2.00–7.00)	3.00 (2.00–6.00)	<0.001
IAND (mmHg)	5.80 (2.70–10.8)	7.30 (3.30–13.9)	6.10 (2.80–11.5)	<0.001
Heart Rate (bmp)	74.2 ± 14.2	75.5 ± 14.7	74.5 ± 14.3	<0.001
SBP (mmHg)	129 ± 18.2	137 ± 20.4	131 ± 19.1	<0.01
DBP (mmHg)	81.5 ± 10.8	83.6 ± 11.4	82.0 ± 11.0	<0.01
Map (mmHg)	96.7 (90.0–105)	100 (93.3–110)	97.3 (90.4–106)	0.142
FBG (mmol/L)	5.67 ± 1.65	6.16 ± 2.14	5.79 ± 1.80	<0.001
BMI (kg/m^2^)	24.9 ± 3.26	25.3 ± 3.37	25.0 ± 3.30	<0.001
LDL-C (mmol/L)	2.72 ± 1.03	2.81 ± 1.06	2.74 ± 1.03	<0.001
HDL-C (mmol/L)	1.47 ± 0.77	1.47 ± 0.72	1.47 ± 0.76	0.635
Triglycerides (mmol/L)	1.29 (0.86–1.96)	1.35 (0.95–2.15)	1.29 (0.89–2.01)	<0.001
hs-CRP (mg/L)	0.90 (0.30–2.00)	1.00 (0.38–2.40)	0.95 (0.31–2.09)	<0.001
UA (µmol/L)	314 ± 94.2	319 ± 94.1	316 ± 94.2	<0.001
eGFR [ml/(min/1.73 m^2^)]	99.1 ± 20.2	95.1 ± 20.8	98.3 ± 20.4	<0.001
Dominant hand (left), *n* (%)	1,620 (5.64)	462 (4.79)	2,082 (5.43)	0.001
Smoking status, *n* (%)				<0.001
Never	18,957 (66.0)	6,340 (65.7)	25,297 (66.0)	
Past	4,677 (16.3)	1,452 (15.1)	6,129 (16.0)	
Current	5,071 (17.7)	1.859 (19.3)	6,930 (18.1)	
Alcohol intake, *n* (%)				0.001
Never	15,074 (52.5)	5,110 (53.0)	20,184 (54.9)	
Past	171 (0.60)	95 (0.98)	266 (0.69)	
Current	8,986 (31.3)	2,986 (30.9)	11,972 (31.2)	
Physical activity, *n* (%)				<0.001
Never	10,385 (36.2)	3,629 (37.6)	14,014 (36.5)	
1–2 times per week	8,030 (28.0)	2,674 (227.7)	10,704 (27.9)	
≥3 times per week	2,456 (8.56)	955 (9.90)	3,411 (8.89)	
Hypertension, *n* (%)	11,118 (38.7)	5,367 (55.6)	16,485 (43.0)	<0.001
Diabetes, *n* (%)	3,500 (12.2)	2,163 (22.4)	5,663 (14.8)	<0.001
Antihypertensive drugs, *n* (%)	3,403 (11.9)	2,007 (20.8)	5,410 (14.1)	<0.001
Antihyperglycemic drugs, *n* (%)	950 (3.31)	700 (7.25)	1,650 (4.30)	<0.001
Lipid-lowering drugs, *n* (%)	178 (0.62)	95 (0.98)	273 (0.71)	<0.001

Values presented are mean ± SD or median (interquartile range).

RbaPWV, right brachial-ankle pulse wave velocity; LbaPWV, left brachial-ankle pulse wave velocity; Rabi, right ankle-brachial index; Labi, left ankle-brachial index.

In the multivariate backward stepwise logistic regression model, the risk factors for a high BPWVD include older age and higher baseline values of baPWV, IAD, BMI, and LDL-C, higher prevalence of diabetes mellitus and intake of antihyperglycemic drugs, lower prevalence of regular physical activities, and lower eGFR at baseline (Supplementary Table S8).

### Survival analysis of the influence of BPWVD on CVD and all-cause mortality

**Figure 2:**
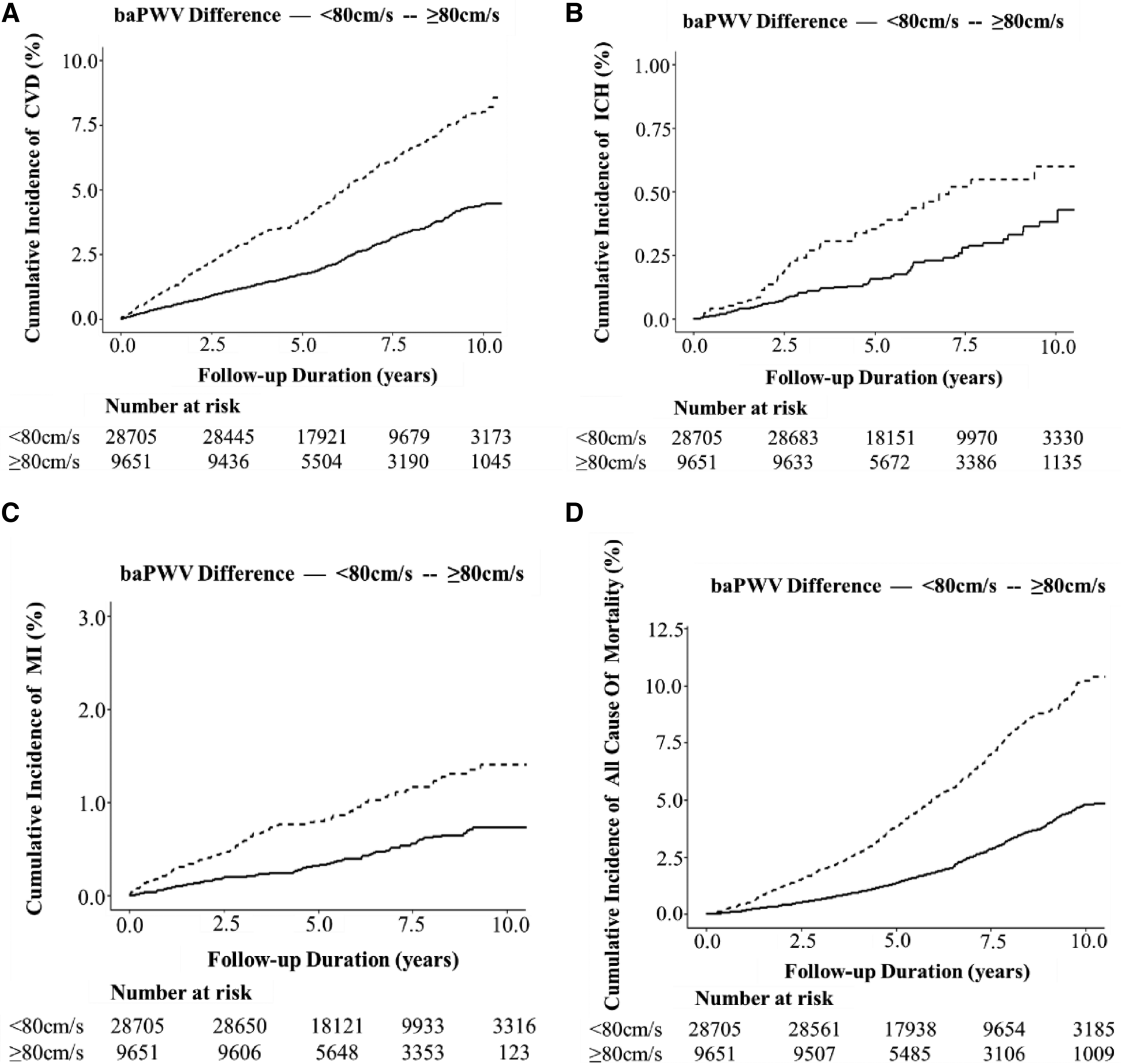
Kaplan –Meier plot of the cumulative incidence of cardiovascular diseases and all-cause mortality over a mean of 6.19 years among 38,356 participants with high or normal bilateral baPWV difference. (**A**) CVD; (**B**) ICH; (**C**) MI; and (**D**) all-cause mortality.

We detected 1,213 CVDs over a mean duration of 6.19 years, including 886 cases of CI, 105 cases of ICH, and 222 cases of MI, along with 1,182 cases of all-cause mortality. The high BPWVD group had statistically (log-rank test) higher cumulative incidence rates for all events when compared with the normal BPWVD group (Table 2) (Figure 2 and Supplementary Figure S3). The Cox proportional hazard model revealed that, following multivariate adjustment, per standard deviation increment in BPWVD was associated with increasing risk for incident CVDs, MI, and all-cause mortality. HRs (95% confidence interval) were 1.06 (1.01–1.11), 1.11 (1.02–1.21), 1.07 (1.04–1.10), respectively (Table 3). In the participants with higher baPWV on the left side, the HRs (95% confidence interval) were 1.08 (1.02–1.14) for CVDs, 1.27 (1.10–1.46) for incident ICH, 1.16 (1.00–1.24) for incident MI, and 1.10 (1.07–1.15) for all-cause mortality, for per standard deviation increment in BPWVD (Table 4). However, no statistically significant association was observed between BPWVD and all events in participants with higher baPWV on the right side (Supplementary Table S9).

**Table 2:** Incidence rates of cardiovascular diseases and all-cause mortality in high or normal bilateral baPWV difference group among 38,356 participants.

	<80 cm/s (*n* = 2,8,705)		≥80 cm/s (*n* = 9,651)		*P*-value^a^
	Event (%)	Incidence rate (per 1,000 person-years)	Event (%)	Incidence rate (per 1,000 person-years)	
CVD	721 (2.51)	4.03 (3.75–4.33)	492 (5.10)	8.40 (7.69–9.18)	<0.001
Cerebral infarction (CI)	530 (1.85)	2.95 (2.71–3.21)	356 (3.69)	6.02 (5.43–6.68)	<0.001
ICH	64 (0.22)	0.35 (0.28–0.45)	41 (0.42)	0.68 (0.50–0.92)	0.001
MI	127 (0.44)	0.70 (0.59–0.84)	95 (0.98)	1.58 (1.30–1.94)	<0.001
All-cause mortality	655 (2.28)	3.65 (3.39–3.95)	527 (5.46)	9.02 (8.28–9.82)	<0.001

^a^ The log-rank test was used for between-group differences in incidence rates.

**Table 3:** Hazard ratios for bilateral baPWV difference related to cardiovascular disease and all-cause mortality among 38,356 participants.

	<80 cm/s (*n* = 28,705)		>80 cm/s (*n* = 9,651)				
	HR (95% confidence interval)		HR (95% confidence interval)				
		Model 1	Model 2	Model 3	Model 4	Per SD	*P*-value
Cardiovascular disease	Ref.	1.32 (1.15–1.52)	1.22 (1.04–1.43)	1.21 (1.03–1.42)	1.09 (0.93–1.29)	1.06 (1.01–1.11)	0.016
Cerebral infarction	Ref.	1.26 (1.07–1.49)	1.16 (0.96–1.40)	1.15 (0.95–1.39)	1.03 (0.85–1.26)	1.02 (0.96–1.09)	0.489
Intracerebral hemorrhage	Ref.	1.26 (0.77–2.05)	1.13 (0.65–1.99)	1.16 (0.66–2.03)	0.96 (0.54–1.73)	1.13 (0.98–1.31)	0.083
Myocardial infarction	Ref.	1.52 (1.11–2.08)	1.43 (1.01–2.04)	1.39 (0.97–1.98)	1.35 (0.94–1.94)	1.11 (1.02–1.21)	0.014
All-cause mortality	Ref.	1.20 (1.07–1.36)	1.18 (1.05–1.34)	1.16 (1.03–1.31)	1.12 (0.99–1.27)	1.07 (1.04–1.10)	<0.001

Per SD, hazard ratio for per standard deviation (78.8 cm/s) change in the bilateral baPWV difference.

Model 1: adjusted age, sex, mean arterial pressure, heart rate, fasting blood glucose, body mass index, low-density lipoprotein cholesterol, high-density lipoprotein cholesterol, log-transformed high-sensitivity C-reactive protein, uric acid, estimated glomerular filtration rate, smoking status, alcohol intake, physical activity, and dominant hand (left). Model 2: Model 1 + lipid-lowering drugs, antihypertensive drugs, and antihyperglycemic drugs. Model 3: Model 2 + inter-arm blood pressure difference, inter-ankle systolic blood pressure difference. Model 4: Model 3 + baseline brachial-ankle pulse wave velocity.

**Table 4:** Hazard ratios for bilateral baPWV difference related to cardiovascular disease and all-cause mortality among 19,421 participants with the left higher baPWV.

	<80 cm/s (*n* = 14,219)		≥80 cm/s (*n* = 5,202)				
	HR (95% confidence interval)		HR (95% confidence interval)				
		Model 1	Model 2	Model 3	Model 4	Per SD	*P*-value
CVD	Ref.	1.34 (1.11–1.61)	1.34 (1.08–1.65)	1.33 (1.07–1.64)	1.16 (0.94–1.45)	1.08 (1.02–1.14)	0.013
Cerebral Infarction (CI)	Ref.	1.24 (1.00–1.56)	1.27 (0.99–1.62)	1.26 (0.98–1.62)	1.10 (0.84–1.42)	1.03 (0.95–1.11)	0.478
ICH	Ref.	1.82 (0.94–3.51)	1.68 (0.81–3.49)	1.67 (0.80–3.47)	1.22 (0.57–2.64)	1.27 (1.10–1.46)	<0.001
MI	Ref.	1.47 (0.99–2.19)	1.40 (0.89–2.20)	1.37 (0.87–2.16)	1.35 (0.84–2.16)	1.16 (1.00–1.24)	0.047
All-cause Mortality	Ref.	1.31 (1.11–1.55)	1.28 (1.08–1.52)	1.25 (1.06–1.48)	1.22 (1.03–1.45)	1.10 (1.07–1.15)	<0.001

Per SD, hazard ratio for per standard deviation (78.8 cm/s) change in the bilateral baPWV difference.

Model 1: adjusted age, sex, mean arterial pressure, heart rate, fasting blood glucose, body mass index, low-density lipoprotein cholesterol, high-density lipoprotein cholesterol, log-transformed high-sensitivity C-reactive protein, uric acid, estimated glomerular filtration rate, smoking status, alcohol intake, physical activity, and dominant hand (left). Model 2: Model 1 + lipid-lowering drugs, antihypertensive drugs, and antihyperglycemic drugs. Model 3: Model 2 + inter-arm blood pressure difference, inter-ankle systolic blood pressure difference. Model 4: Model 3 + baseline brachial-ankle pulse wave velocity.

For sensitivity analyses, the results among men were similar to those in the overall population, with an HR (95% confidence interval) of 1.06 (1.01–1.11) for incident CVDs, 1.13 (1.04–1.23) for MI, and 1.07 (1.04–1.10) for all-cause mortality. There was no significance observed in women (Supplementary Table S10). In addition, after excluding participants with an ABI ≤ 0.9 or IAD ≥ 15mmHg, respectively, the HRs (95% confidence interval) in participants without ABI ≤ 0.9 were 1.19 (1.03–1.37) for incident ICH and 1.07 (1.02–1.12) for all-cause mortality (Supplementary Table S11). The HRs (95% confidence interval) in participants without IAD ≥15 mmHg were 1.07 (1.02–1.13) for incident CVD, 1.17 (1.03–1.33) for incident ICH, 1.11 (1.02–1.10) for MI, and 1.07 (1.04–1.10) for all-cause mortality (Supplementary Table S12). Furthermore, for participants without ABI ≤ 0.9 and IAD ≥ 15 mmHg, the HRs (95% confidence interval) were 1.21 (1.06–1.40) for incident ICH and 1.07 (1.02–1.12) for all-cause mortality (Supplementary Table S13). As the covariates vary over time, we transformed the covariates into updated confounders and further conducted time-dependent covariate analysis (Supplementary Table S14). Considering the influence of hypertension, diabetes, and overweight/obesity on the measurement of baPWV, we investigated the impact of BPWVD on outcomes under different blood pressure conditions, different blood glucose levels, and overweight/obesity conditions. The results indicated that participants with hypertension, diabetes, and overweight/obesity combined with BPWVD value of ≥80 cm/s had a higher risk of developing CVD and all-cause mortality (Supplementary Tables S15–S18). In order to further validate the reliability of the results, we conducted propensity scoring, and the results were similar (Supplementary Table S19).

We compared the predictive value of BPWVD, ABI, and IAD in this population. The discriminatory power to identify CVDs ranged from 0.62 to 0.73 using the BPWVD method, while the ability to identify all-cause mortality ranged from 0.66 to 0.76 using the same method. Both ABI (AUC for CVDs: from 0.51 to 0.50, AUC for all-cause mortality: from 0.50 to 0.50) and IAD to CVD (AUC for CVDs: from 0.45 to 0.36, AUC for all-cause mortality: from 0.48 to 0.40) did not provide significantly predictive value in the study population (Supplementary Table S20 and Supplementary Figures S4,S5).

## Discussion

We defined the high BPWVD group by using a threshold value of 80 cm/s. The study findings indicate that while no significant increase in the risk for new-onset cardiovascular disease and all-cause mortality was observed in the high BPWVD group, an increase of 1 SD in BPWVD was associated with 6%, 11%, and 7% increased risks of CVD, MI, and all-cause mortality, respectively. Furthermore, participants with elevated BPWVD and higher left baPWV values had a 22% greater risk of all-cause death compared with those with normal BPWVD values after adjusting for baseline baPWV and all confounding factors. In addition, for each standard deviation increase in BPWVD, the risk for CVD, ICH, MI, and all-cause mortality increased by 8%, 27%, 16%, and 10%, correspondingly. After excluding the cases with IAD ≥ 10 mmHg or ABI ≥ 15 mmHg, similar results were found. These findings may indicate the following: (1) independent of baseline baPWV and conventional risk factors, an elevated BPWVD could be a risk factor for CVD, MI, ICH and all-cause mortality, and it might provide a new clinical marker for screening high-risk populations for CVD, MI, ICH, and all-cause mortality; (2) the increased risk of CVD and all-cause mortality caused by BPWVD may have a dose–response relationship, and the optimal cut-off value needs to be further explored; and (3) this bilateral baPWV difference might be more representative when the left side baPWV value is higher than the right side.

Several studies have demonstrated that ABI, IAD, and baPWV are associated with an increased risk of incident CVD and death. Tokitsu et al. (21) reported that increased IAD was positively associated with a higher likelihood of experiencing coronary events in the future. Criqui et al. (22) observed that lower ABI was associated with an increased risk of CVD in individuals without known history of CVDs. One study found that increasing carotid-femoral PWV could raise the risk of incident coronary heart disease by 2.07 times (23); higher baPWV would increase the risk of coronary-related events in patients with heart failure or with preserved left ventricular ejection fraction (24). Therefore, the association between BPWVD and CVD, MI, and all-cause mortality may be reasonable. Our results prove that BPWVD is superior to ABI and IAD in predicting these outcomes, as evidenced by larger AUCs in this study. Previous studies have also reported that both lower ABI and higher PWV have been associated with the occurrence of microbleeds and intracerebral hemorrhage (25, 26). In the absence of peripheral artery disease, higher BPWVD may, by unevenly altering wall thickness and elasticity, lead to greater fluctuation in the pressure of blood flow, thus increasing the risk of intracerebral hemorrhage. This suggested a potential independent association between BPWVD and the cerebral vascular health.

Although we found positive associations between higher BPWVD and higher incidence of CVDs, ICH, MI, and all-cause mortality, the underlying mechanisms remain largely unclear. One plausible explanation is that arterial stiffness is more severe on one side than on the other side. First, bilateral baPWV measurements originate anatomically from the same arterial vessel, the aorta, through which arterial blood flow diverges into the peripheral arteries. Thus, the difference in baPWV between the left and right sides may result from variations of arterial stiffness in four extremities, despite them originating from the same vascular, the aorta. Second, an abnormally high BPWVD could be caused by an increase in baPWV on one side or a decrease in baPWV on the other side. After excluding participants with an abnormally low ABI of ≤ 0.9 or an abnormally high IAD of ≥ 15 mmHg, our findings remained statistically significant. Therefore, a drop in baPWV on one side may not be the primary reason for the finding (4). The findings derived from our investigation on the participants with a baPWV higher on the left side supported this hypothesis. In contrast to the right subclavian artery, the left subclavian artery leaves the aorta at a narrower angle, potentially causing an increased velocity of blood flow on the left side. In addition, previous research (27, 28) has demonstrated the existence of a gradient in arterial stiffness between the aorta and the arteries in the upper limb. This observation suggests that there may be variations in arterial stiffness between the left and right sides, indicating a possible association with disease occurrence.

Our findings have important clinical implications for the management and prevention of CVDs. As a cost-effective, easily accessible, and non-invasive clinical parameter, BPWVD should be considered during baPWV measurements. Using only one side for the baPWV measurements may result in either overestimations or underestimations of arterial stiffness. In a study conducted by Motobe et al., it was shown that after excluding the subjects with arteriosclerosis obliterans (ASOs), a low ABI and a borderline ABI measured by the Vascular Profiler are frequently observed in healthy young individuals in Japan (9). The prevalence of ABI ≤ 0.9 may lead to a lower baPWV value than the normal value, and ABI ≥ 1.33 might lead to a higher baPWV value than the normal value (29–31.) Therefore, it is more sensible to use the mean baPWV value on both sides to represent arterial stiffness status. Individuals with normal PWV values but high BPWVD may still lack awareness of the potential hazards and overlook the associated cardiovascular risks. Consequently, these findings emphasize the importance of adopting a more proactive approach to population screening for high BPWVD and implementing more intensive interventions to modify cardiovascular risk factors among individuals with high BPWVD.

To the best of our knowledge, this was the first large-scale study reporting the association of BPWVD with CVD and all-cause mortality through baPWV measurements. The bilateral baPWV measurements were performed simultaneously following standard protocols to avoid measurement errors. We further adjusted detailed demographics, lifestyle factors, and biochemical parameters in the statistical models and incorporated the consideration of the dominant hand (left/right) in our analysis.

Several limitations of our study also merit consideration. First, our study is restricted to one cohort, with a relatively short follow-up period. Thus, it is necessary to validate our findings by conducting future analyses that involve additional demographic or ethnic populations with longer follow-up intervals. Second, participants with an ABI ≤ 0.9 and an IAD ≥ 15 mmHg were excluded from medical imaging examinations, as these procedures are widely recognized as the gold standard for diagnosing peripheral artery disease. Third, we arbitrarily used the upper quartile of the BPWVD as the cut-off value for the classification of the “high BPWVD group,” so that future studies may assess the best-separating cut-off value for the definition of the high BPWVD group. Fourth, we measured arterial stiffness using the baPWV instead of the carotid-femoral PWV. Nonetheless, various studies have reported a robust correlation between the carotid-femoral PWV and the baPWV. The American Heart Association has recognized baPWV as a widely used and clinically accepted measure for determining arterial stiffness, with a rating of “Class I, Level of Evidence B” (2). Fifth, we do not yet have a good explanation of the pathogenesis of intracerebral hemorrhage, thus warranting further investigation in future research. Whether the difference in the dominant hand would be associated with the localization of intracerebral hemorrhage (left/right) is a subject that warrants further investigation in future studies that encompass a larger sample size.

## Conclusion

An elevated BPWVD can be an independent risk factor for CVD, MI, ICH, and all-cause mortality, which remains significant even after adjusting for baseline baPWV and traditional risk factors. This finding is significant and suggests that BPWVD has the potential to be a novel clinical marker for identifying high-risk populations. Moreover, the increased risk associated with BPWVD may exhibit a dose–response relationship with CVD and all-cause mortality. However, the optimal cut-off value needs to be further explored. These findings indicate that in addition to focusing on the value of baPWV during the baPWV measurement, we also need to pay more attention to the difference in arterial stiffness between the two sides, which may serve as a new indicator for identifying individuals at high risk for cardiovascular complications.

## Data Availability

The datasets presented in this article are not readily available due to restrictions. Requests to access the datasets should be directed to the corresponding author.
