# Supplementary material for: The impact of bilateral brachial-ankle pulse wave velocity difference on cardiovascular disease and all-cause mortality
Source: Front Cardiovasc Med. 2023 Oct 6;10:1234325. doi: 10.3389/fcvm.2023.1234325 (PMC10588177; doi:10.3389/fcvm.2023.1234325)
Supplement: Supplementary file 1 [file Table1.doc]

**Supplementary data for “**Elevated Bilateral Pulse Wave Velocity Difference Associated with Incident Cardiovascular Disease and All-Cause Mortality**”**

**Content list:**

**Table S1.** Baseline comparison of included participants versus excluded participants.

**Table S2.** The baseline characteristics of the 77 subjects for assessing the reproducibility of baPWV and BPWVD.

**Table S3.** The baPWV, ABI, blood pressure and heart rate values in three repeated measurements among 77 participants.

**Table S4**. Correlation of different PWV values in repeated measurements.

**Table S5.** Variance allocation of different baPWV values in three repeated measurements. (n=77)

**Table S6.** Variance allocation of different PWV values in last two measurements. (n=77)

**Table S7.** Baseline characteristics of participants with higher baPWV values on the left or right side.

**Table S8.** The multivariable analysis of relationship of conventional factors and bilateral baPWV difference.

**Table S9.** Hazard ratios for bilateral baPWV difference related to cardiovascular disease and all-cause mortality among 18,935 participants with the right higher baPWV.

**Table S10.** Hazard ratios for bilateral baPWV difference related to cardiovascular disease and all-cause mortality among 38,356 participants in difference gender group.

**Table S11.** Hazard ratios for bilateral baPWV difference related to cardiovascular disease and all-cause mortality among 36,780 participants after excluding participants with ABI≤0.9.

**Table S12.** Hazard ratios for bilateral baPWV difference related to cardiovascular disease and all-cause mortality among 36,353 participants after excluding participants with IAD≥15mmHg.

**Table S13.** Hazard ratios for bilateral baPWV difference related to cardiovascular disease and all-cause mortality among 35,053 participants after excluding participants with ABI≤0.9 or IAD≥15mmHg.

**Table S14.** Time-dependent analysis for bilateral baPWV difference related to cardiovascular disease and all-cause mortality among 38,356 participants.

**Table S15.** Hazard ratios (95% confidence interval) of cardiovascular disease and all-cause mortality in groups stratified by the bilateral difference in the brachial-ankle pulse wave velocity (BPWVD) and blood pressure status (n=38,356).

**Table S16.** Hazard ratios of cardiovascular disease and all-cause mortality in groups stratified by the bilateral difference in the brachial-ankle pulse wave velocity (BPWVD) and different fasting blood glucose concentration status (n=38,356).

**Table S17.** Hazard ratios of cardiovascular disease and all-cause mortality in groups stratified by the bilateral difference in the brachial-ankle pulse wave velocity (BPWVD) and overweight status (n=38,356)

**Table S18**. Hazard ratios of cardiovascular disease and all-cause mortality in groups stratified by the bilateral difference in the brachial-ankle pulse wave velocity (BPWVD) and obesity status (n=38,356)

**Table S19.** Hazard ratios for bilateral baPWV difference related to cardiovascular disease and all-cause mortality after performing propensity score matching with consideration of several important factors including age, sex, systolic blood pressure, and heart rate.

**Table S20.** Time-dependent area under the receiver operating characteristic curve for cardiovascular diseases and all-cause mortality by BPWVD, ABI and IAD.

**Figure S1.** Follow chart.

**Figure S2.** System for measuring baPWV.

**Figure S3.** Kaplan-Meier plot of the cumulative incidence of Cerebral Infarction over a mean of 6.19 years among 38,356 participants with high or normal bilateral baPWV difference.

**Figure S4.** ROC curves for cardiovascular diseases by BPWVD, ABI and IAD at 2, 4, 6, 8 and 10 years by using the Aalen method.

**Figure S5.** ROC curves for all-cause mortality by BPWVD, ABI and IAD at 2, 4, 6, 8 and 10 years by using the Aalen method.

Table S1. Baseline comparison of included participants versus excluded participants.

|  | Included population (n=38,356) | Excluded due to not participating/ incomplete information population (n=2,483) | P value |
| --- | --- | --- | --- |
| Age (years) | 48.4 ± 12.7 | 41.7 ± 14.7 | <0.001 |
| Sex, n (%) | 27,691 (72.5) | 1016 (40.9) | <0.001 |
| Difference of baPWV (cm/s) | 42.0 (19.0, 80.0) | 42.0 (19.0, 78.0) | 0.705 |
| RbaPWV (cm/s) | 1,477 ± 325 | 1,425 ± 326 | <0.001 |
| LbaPWV (cm/s) | 1,482 ± 332 | 1,428 ± 330 | <0.001 |
| Rabi | 1.11 (1.05, 1.18) | 1.10 (1.04, 1.18) | 0.201 |
| Labi | 1.11 (1.05, 1.18) | 1.10 (1.04, 1.17) | 0.044 |
| IAD (mmHg) | 3.00 (2.00, 6.00) | 3.00 (2.00, 6.00) | 0.110 |
| IAND (mmHg) | 6.10 (2.80, 11.5) | 6.30 (2.90, 12.0) | 0.613 |
| Heart Rate (bmp) | 74.5 ± 14.3 | 73.6 ± 10.7 | 0.025 |
| SBP (mmHg) | 131 ± 19.1 | 126 ± 19.6 | <0.001 |
| DBP (mmHg) | 82.0 ± 11.0 | 81.8 ± 11.5 | 0.449 |
| Map (mmHg) | 97.3 (90.4, 106) | 94.4 (87.8, 104) | 0.409 |
| FBG (mmol/L) | 5.79 ± 1.80 | 5.43 ± 1.58 | <0.001 |
| BMI (kg/m2) | 25.0 ± 3.30 | 24.5 ± 3.55 | <0.001 |
| LDL-C (mmol/L) | 2.74 ± 1.03 | 2.56 ± 0.81 | <0.001 |
| HDL-C (mmol/L) | 1.47 ± 0.76 | 1.47 ± 0.40 | 0.986 |
| Triglycerides (mmol/L) | 1.29 (0.89, 2.01) | 1.21 (0.81, 1.84) | 0.003 |
| Hs-CRP (mg/L) | 0.95 (0.31, 2.09) | 0.70 (0.10, 1.70) | 0.111 |
| UA (µmol/L) | 316 ± 94.2 | 302 ± 91.8 | <0.001 |
| eGFR [mL/(min·1.73m2)] | 98.3 ± 20.4 | 91.1 ± 20.9 | <0.001 |
| Dominant hand (Left), n (%) | 2,082 (5.43) | 3 (0.12) | <0.001 |
| Smoking status, n (%) |  |  | <0.001 |
| Never | 25,297 (66.0) | 823 (33.2) |  |
| Past | 6,129 (16.0) | 61 (2.46) |  |
| Current | 6,930 (18.1) | 453 (18.2) |  |
| Alcohol intake, n (%) |  |  | <0.001 |
| Never | 20,184 (54.9) | 689 (27.8) |  |
| Past | 266 (0.69) | 35 (1.41) |  |
| Current | 11,972 (31.2) | 611 (24.6) |  |
| Physical activity, n (%) |  |  | <0.001 |
| Never | 14,014 (36.5) | 243 (9.79) |  |
| 1–2 times per week | 10,704 (27.9) | 944 (38.0) |  |
| ≥3 times per week | 3,411 (8.89) | 140 (5.64) |  |
| Hypertension, n (%) | 16,485 (43.0) | 478 (19.3) | <0.001 |
| Diabetes, n (%) | 5,663 (14.8) | 93 (3.75) | <0.001 |
| Antihypertensive drugs, n (%) | 5,410 (14.1) | 133 (5.36) | <0.001 |
| Antihyperglycemic drugs, n (%) | 1,650 (4.30) | 30 (1.21) | <0.001 |
| Lipid-lowering drugs, n (%) | 273 (0.71) | 12 (0.48) | <0.001 |

Values presented are mean ± SD or median (interquartile range).

Abbreviations: RbaPWV, right brachial ankle pulse wave velocity; LbaPWV, left brachial ankle pulse wave velocity; Rabi, right ankle brachial index; Labi, left ankle brachial index; IAD, inter-arm difference; IAND, inter-ankle systolic blood pressure difference; SBP, systolic blood pressure; DBP, diastolic blood pressure MAP, mean arterial pressure; FBG, fasting blood glucose; BMI, body mass index, LDL-C, low-density lipoprotein cholesterol; HDL-C, high density lipoprotein cholesterol; hs-CRP, high-sensitivity C-reactive protein; UA, uric acid; eGFR, estimated glomerular filtration rate.

Table S2. The baseline characteristics of the 77 subjects for assessing the reproducibility of baPWV and BPWVD.

| [Variable](javascript:;) | Value |
| --- | --- |
| Age (years) | 52.0 ± 16.1 |
| Male, n (%) | 33 (42.9) |
| Body mass index (kg/m2) | 25.0 ± 3.86 |
| Waist (cm) | 77.4 ± 26.2 |
| Hypertension, n (%) | 23 (30.0) |
| Diabetes, n (%) | 10 (13.0) |
| [Hyperlipidemia](javascript:;), n (%) | 4 (5.19) |

Table S3. The baPWV, ABI, blood pressure and heart rate values in three repeated measurements among 77 participants.

| [Variable](javascript:;) | First measurement | Second measurement | Third measurement | P value^*^ |
| --- | --- | --- | --- | --- |
| RbaPWV (cm/s) | 1386 ± 302 | 1399 ± 318 | 1399 ± 291 | 0.16 |
| LbaPWV (cm/s) | 1394 ± 329 | 1410 ± 344 | 1415 ± 323 | 0.04 |
| Rabi | 1.12 ± 0.09 | 1.12 ± 0.08 | 1.10 ± 0.08 | 0.02 |
| Labi | 1.10 ± 0.09 | 1.10 ± 0.08 | 1.09 ± 0.08 | 0.01 |
| RbSBP (mmHg) | 123 ± 17.9 | 123 ± 17.8 | 126 ± 19.2 | <0.01 |
| LbSBP (mmHg) | 121 ± 16.1 | 122 ± 16.6 | 124 ± 17.8 | <0.01 |
| Heart rate (bmp) | 67.5 ± 16.3 | 68.8 ± 17.6 | 64.6 ± 21.7 | 0.05 |
| BPWVD (cm/s) | 39.0 (21.0,82.0) | 40.0 (22.0,81.0) | 41.0 (20.0,70.0) | 0.07 |

^*^ P value was used to test differences between any two groups.

Abbreviations: RbaPWV, right brachial ankle pulse wave velocity; LbaPWV, left brachial ankle pulse wave velocity; Rabi, right ankle brachial index; Labi, left ankle brachial index; RbSBP, right brachial systolic blood pressure; LbSBP, left brachial systolic blood pressure; BPWVD, bilateral difference in brachial-ankle pulse wave velocity.

Table S4. Correlation of different PWV values in repeated measurements.

|  | First vs Second | | First vs Third | | Second vs Third | |
| --- | --- | --- | --- | --- | --- | --- |
|  | β | P value | β | P value | β | P value |
| RbaPWV (cm/s) | 0.99 | <0.01 | 0.97 | <0.01 | 0.98 | <0.01 |
| LbaPWV (cm/s) | 0.99 | <0.01 | 0.98 | <0.01 | 0.98 | <0.01 |
| Rabi | 0.83 | <0.01 | 0.78 | <0.01 | 0.85 | <0.01 |
| Labi | 0.87 | <0.01 | 0.81 | <0.01 | 0.83 | <0.01 |
| RbSBP (mmHg) | 0.95 | <0.01 | 0.96 | <0.01 | 0.96 | <0.01 |
| LbSBP (mmHg) | 0.94 | <0.01 | 0.94 | <0.01 | 0.95 | <0.01 |
| BPWVD (cm/s) | 0.69 | <0.01 | 0.47 | <0.01 | 0.63 | <0.01 |

Abbreviations: RbaPWV, right brachial ankle pulse wave velocity; LbaPWV, left brachial ankle pulse wave velocity; Rabi, right ankle brachial index; Labi, left ankle brachial index; RbSBP, right brachial systolic blood pressure; LbSBP, left brachial systolic blood pressure; BPWVD, bilateral difference in brachial-ankle pulse wave velocity.

Table S5. Variance allocation of different baPWV values in three repeated measurements. (n=77)

|  | RbaPWV (cm/s) | LbaPWV (cm/s) | BPWVD (cm/s) |
| --- | --- | --- | --- |
| ICC | 0.98 | 0.98 | 0.58 |
| AIC a | 2790 | 2768 | 2391 |
| Between persons σ2 (%) b | 90095 (0.98) | 108425 (0.98) | 1578 (0.58) |
| Between persons σ2 (95% CI) c | (66976, 127717) | (80666, 153534) | (1099,2452) |
| Within person σ2 (%) b | 2270 (0.02) | 1793(0.02) | 1125 (0.42) |
| Within person σ2 (95% CI) c | (1835, 2881) | (1449, 2275) | (910, 1428) |

Abbreviations: ICC, Intraclass correlation coefficients; σ^2^ = variance. baPWV, brachial-ankle pulse wave velocity.

*^a^*Akaike information criterion (AIC) values were used to compare the fit of models.

^b^The proportion of between-person variance to the total variance.

^c^Confidence intervals for the variance components.

Table S6. Variance allocation of different PWV values in last two measurements. (n=77)

|  | RbaPWV (cm/s) | LbaPWV (cm/s) | BPWVD (cm/s) |
| --- | --- | --- | --- |
| ICC | 0.97 | 0.98 | 0.63 |
| AIC a | 1958 | 1960 | 1578 |
| Between persons σ2 (%) b | 90528 (0.97) | 109247 (0.98) | 1402 (0.63) |
| Between persons σ2 (95% CI) c | (67205, 128577) | (81188, 154935) | (957,2250) |
| Within person σ2 (%) b | 2428 (0.03) | 2084 (0.02) | 828 (0.37) |
| Within person σ2 (95% CI) c | (1809, 3430) | (1553, 2945) | (617, 1171) |

Abbreviations: ICC, Intraclass correlation coefficients; σ2 = variance.

aAkaike information criterion (AIC) values were used to compare the fit of models.

bThe proportion of between-person variance to the total variance.

^c^Confidence intervals for the variance components.

Table S7. Baseline characteristics of participants with higher baPWV values on the left or right side.

|  | Right ≥ Left | | Left > Right | |
| --- | --- | --- | --- | --- |
|  | <80cm/s (n=14,486) | ≥80cm/s (n=4,449) | <80cm/s  (n=14,219) | ≥80cm/s  (n=5,202) |
| Age (years) | 46.9 ± 11.9 | 51.4 ± 13.9 | 47.2 ± 12.0 | 53.5 ± 14.0 |
| Sex, n (%) | 10,567 (73.2) | 3,507 (79.0) | 9,788 (69.1) | 3,829 (74.0) |
| Difference of baPWV (cm/s) | 29.0 (14.0, 49.0) | 117 (94.0, 158) | 30.0 (14.0,50.0) | 122 (96.0,173) |
| RbaPWV (cm/s) | 1,441 ± 275 | 1,680 ± 393 | 1,410 ± 284 | 1,588 ± 394 |
| LbaPWV (cm/s) | 1,409 ± 274 | 1,533 ± 363 | 1,444 ± 286 | 1,745 ± 423h |
| Rabi | 1.11 (1.05, 1.18) | 1.12 (1.05, 1.18) | 1.11 (1.05, 1.17) | 1.11 (1.04, 1.18) |
| Labi | 1.12 (1.05, 1.18) | 1.11 (1.04, 1.18) | 1.11 (1.05, 1.17) | 1.11 (1.04, 1.18) |
| IAD (mmHg) | 3.00 (1.00, 6.00) | 4.00 (2.00, 7.00) | 3.00 (1.00, 6.00) | 4.00 (2.00, 7.00) |
| AAD (mmHg) | 5.90 (2.70, 11.0) | 7.60 (3.40, 14.0) | 5.70 (2.60, 10.6) | 7.00 (3.20,13.8) |
| Heart Rate (bmp) | 74.1 ± 13.7 | 75.4 ± 15.0 | 74.3 ± 14.7 | 75.7 ± 14.4 |
| SBP (mmHg) | 130 ± 18.0 | 137 ± 20.2 | 129 ± 18.4 | 138 ±20.6 |
| DBP (mmHg) | 81.7 ± 10.8 | 83.7 ± 11.3 | 81.3 ± 10.8 | 83.5 ± 11.4 |
| Map (mmHg) | 97.0 (90.0, 105) | 100 (93.3, 109) | 96.7 (89.8,104) | 100 (93.3,110) |
| FBG (mmol/L) | 5.69 ± 1.65 | 6.15 ± 2.20 | 5.66 ± 1.66 | 6.16 ± 2.08 |
| BMI (kg/m2) | 25.0 ± 3.25 | 25.5 ± 3.38 | 24.8 ± 3.27 | 25.2 ± 3.36 |
| LDL-C (mmol/L) | 2.74 ± 1.01 | 2.82 ± 0.95 | 2.70 ± 1.05 | 2.80 ± 1.14 |
| HDL-C (mmol/L) | 1.47 ± 0.87 | 1.46 ± 0.90 | 1.48 ± 0.67 | 1.47 ± 0.53 |
| Triglycerides (mmol/L) | 1.29 (0.88,2.00) | 1.38 (0.97, 2.18) | 1.27 (0.85, 1.91) | 1.33 (0.93,2.12) |
| Hs-CRP (mg/L) | 0.93 (0.30, 2.00) | 1.09 (0.40, 2.40) | 0.90 (0.30, 1.94) | 1.00 (0.33,2.40) |
| UA (µmol/L) | 317 ± 94.5 | 324 ± 95.5 | 312 ± 93.8 | 315 ± 92.8 |
| eGFR [mL/(min·1.73m2)] | 99.3 ± 20.2 | 96.0 ± 21.3 | 99.3 ± 20.2 | 94.3 ± 20.4 |
| Dominant hand (Left), n (%) | 829 (5.72) | 234 (5.26) | 791 (5.56) | 228 (4.38) |
| Smoking status, n (%) |  |  |  |  |
| Never | 9,443 (65.2) | 2,828 (63.7) | 9,514 (66.9) | 3,512 (67.5) |
| Past | 2,421 (16.7) | 748 (16.8) | 2,256 (15.9) | 704 (13.5) |
| Current | 2,622 (18.1) | 873 (19.6) | 2,449 (17.2) | 986 (19.0) |
| Alcohol intake, n (%) |  |  |  |  |
| Never | 7,416 (51.2) | 2,256 (50.7) | 7,658 (53.9) | 2,854 (54.9) |
| Past | 82 (0.57) | 47 (1.06) | 89 (0.63) | 48 (0.92) |
| Current | 4,693 (32.4) | 1,444 (32.5) | 4,293 (30.2) | 1,542 (29.6) |
| Physical activity, n (%) |  |  |  |  |
| Never | 5,214 (36.0) | 1,687 (37.9) | 5,171 (36.4) | 1,942 (37.3) |
| 1–2 times per week | 3,969 (27.4) | 1,185 (26.4) | 4061 (28.6) | 1,489 (28.6) |
| ≥3 times per week | 1,192 (8.23) | 399 (8.97) | 1,264 (8.89) | 556 (10.7) |
| Hypertension, n (%) | 5,676 (39.2) | 2,425 (54.5) | 5,442 (38.3) | 2,942 (56.6) |
| Diabetes, n (%) | 1,783 (12.3) | 949 (21.3) | 1,717 (12.1) | 1,214 (23.3) |
| Antihypertensive drugs, n (%) | 1,732 (12.0) | 869 (19.5) | 1,671 (11.8) | 1,138 (21.9) |
| Antihyperglycemic drugs, n (%) | 473 (3.27) | 307 (6.90) | 477 (3.35) | 393 (7.55) |
| Lipid-lowering drugs, n (%) | 90 (0.62) | 38 (0.85) | 88 (0.62) | 57 (1.10) |

Values presented are mean±SD or median (interquartile range).

Abbreviations: RbaPWV, right brachial ankle pulse wave velocity; LbaPWV, left brachial ankle pulse wave velocity; Rabi, right ankle brachial index; Labi, left ankle brachial index; IAD, inter-arm difference; IAND, inter-ankle systolic blood pressure difference; SBP, systolic blood pressure; DBP, diastolic blood pressure MAP, mean arterial pressure; FBG, fasting blood glucose; BMI, body mass index, LDL-C, low-density lipoprotein cholesterol; HDL-C, high density lipoprotein cholesterol; hs-CRP, high-sensitivity C-reactive protein; UA, uric acid; eGFR, estimated glomerular filtration rate.

Table S8. The multivariable analysis of relationship of conventional factors and bilateral baPWV difference.

|  | Standard β | OR (95 % CI) | P value |
| --- | --- | --- | --- |
| Age (years) | 0.040 | 1.006 (1.003-1.008) | <0.001 |
| Baseline baPWV(cm/s) | 0.305 | 1.002 (1.002-1.002) | <0.001 |
| IAD (mmHg) | 0.023 | 1.003 (1.002-1.005) | <0.001 |
| BMI (kg/m^2^) | 0.042 | 1.023 (1.016-1.031) | <0.001 |
| LDL-C (mmol/L) | 0.013 | 1.022 (1.000-1.045) | 0.045 |
| eGFR [mL/(min·1.73m2)] | -0.019 | 0.998 (0.997-1.000) | 0.012 |
| ≥3 times per week | -0.017 | 0.992 (0.985-0.998) | 0.016 |
| Diabetes mellitus | 0.035 | 1.197 (1.120-1.280) | <0.001 |

Multivariate logistic regression analysis was performed by using stepwise regression.

Abbreviations: baPWV, brachial ankle pulse wave velocity; BMI, body mass index; LDL-C, low-density lipoprotein cholesterol; eGFR, estimated glomerular filtration rate.

Note: Based on whether the difference in bilateral baPWV is greater than or equal to 80 cm/s (<80=0, ≥80=1) as the dependent variable, age, gender (female=0, male=1), Inter-arm blood pressure difference, inter-ankle systolic blood pressure difference; baseline baPWV, heart rate, body mass index, low density lipoprotein cholesterol, high density lipoprotein cholesterol, log-transformed high-sensitivity C-reactive protein, uric acid, estimated glomerular filtration rate, smoking status (never=0, previous=1, current=2), alcohol intake (never=0, previous=1, current=2), physical activity (no physical activity=0, 1-2 times per week=1, ≥3 times per week=2), dominant hand (right=0, left=1), lipid-lowering drugs (no=0, yes=1), antihypertensive drugs(no=0, yes=1), antihyperglycemic drugs(no=0, yes=1), hypertension(no=0, yes=1), and diabetes mellitus (no=0, yes=1) were used as independent variables.

Table S9. Hazard ratios for bilateral baPWV difference related to cardiovascular disease and all-cause mortality among 18,935 participants with the right higher baPWV.

|  | <80cm/s (n=14,486) |  | ≥80cm/s (n=4,449) | | |  |  |
| --- | --- | --- | --- | --- | --- | --- | --- |
|  | HR (95%CI) |  |  | HR (95%CI) |  |  |  |
|  |  | Model 1 | Model 2 | Model 3 | Model 4 | Per SD | P value |
| Cardiovascular Disease (CVD) | Ref. | 1.27 (1.03-1.57) | 1.03 (0.82-1.33) | 1.02 (0.79-1.31) | 0.97 (0.75-1.25) | 1.03 (0.95-1.12) | 0.510 |
| Cerebral Infarction (CI) | Ref. | 1.27 (0.99-1.63) | 1.01 (0.75-1.35) | 0.99 (0.74-1.33) | 0.93 (0.69-1.26) | 1.02 (0.92-1.12) | 0.772 |
| Intracerebral Hemorrhage (CH) | Ref. | 0.79 (0.36-1.71) | 0.58 (0.22-1.50) | 0.62 (0.24-1.62) | 0.61 (0.23-1.63) | 0.75 (0.43-1.30) | 0.308 |
| Myocardial Infarction (MI) | Ref. | 1.52 (0.91-2.53) | 1.37 (0.78-2.42) | 1.30 (0.73-2.33) | 1.26 (0.70-2.28) | 1.10 (0.94-1.29) | 0.219 |
| All Cause of Mortality | Ref. | 1.09 (0.91-1.30) | 1.08 (0.91-1.29) | 1.07 (0.89-1.27) | 1.02 (0.85-1.22) | 1.04 (0.99-1.08) | 0.131 |

baPWV, brachial-ankle pulse wave velocity.

Per SD, hazard ratio for per standard deviation (78.8 cm/s) change in the bilateral baPWV difference;

Model 1: adjusted age, sex, mean arterial pressure, heart rate, fasting blood glucose, body mass index, low density lipoprotein cholesterol, high density lipoprotein cholesterol, log-transformed high-sensitivity C-reactive protein, uric acid, estimated glomerular filtration rate, smoking status, alcohol intake, physical activity and dominant hand (Left).

Model 2: Model 1 + lipid-lowering drugs, antihypertensive drugs and antihyperglycemic drugs;

Model 3: Model 2+ Interarm blood pressure difference, inter-ankle systolic blood pressure difference;

Model 4: Model 3 + baseline brachial-ankle pulse wave velocity

Table S10. Hazard ratios for bilateral baPWV difference related to cardiovascular disease and all-cause mortality among 38,356 participants in difference gender group.

|  | <80cm/s (n=28,705) |  | >80cm/s (n=9,651) | | |  |
| --- | --- | --- | --- | --- | --- | --- |
|  | HR (95%CI) |  |  | HR (95%CI) |  |  |
|  |  | Model 1 | Model 2 | Model 3 | Per SD | P value |
| Man (n=27,691) |  |  | | | | |
| Cardiovascular Disease (CVD) | Ref. | 1.26 (1.06-1.50) | 1.25 (1.04-1.49) | 1.14 (0.95-1.36) | 1.06 (1.00-1.11) | 0.041 |
| Cerebral Infarction (CI) | Ref. | 1.14 (0.93-1.41) | 1.14 (0.92-1.41) | 1.02 (0.82-1.27) | 1.01 (0.94-1.08) | 0.773 |
| Intracerebral Hemorrhage (CH) | Ref. | 1.19 (0.63-2.24) | 1.21 (0.64-2.29) | 1.01 (0.52-1.95) | 1.06 (0.87-1.31) | 0.550 |
| Myocardial Infarction (MI) | Ref. | 1.69 (1.15-2.46) | 1.63 (1.11-2.39) | 1.63 (1.10-2.41) | 1.13 (1.04-1.23) | 0.003 |
| All Cause of Mortality | Ref. | 1.20 (1.05-1.37) | 1.18 (1.03-1.35) | 1.12 (0.98-1.29) | 1.07 (1.04-1.10) | <0.001 |
| Woman (n=10,527) |  |  |  |  |  |  |
| Cardiovascular Disease (CVD) | Ref. | 1.94 (0.75-1.60) | 1.94 (0.74-1.60) | 1.03 (0.70-1.50) | 1.11 (1.01-1.22) | 0.034 |
| Cerebral Infarction (CI) | Ref. | 1.16 (0.75-1.79) | 1.16 (0.75-1.79) | 1.09 (0.70-1.69) | 1.09 (0.97-1.21) | 0.141 |
| Intracerebral Hemorrhage (CH) | Ref. | 1.29 (0.37-4.50) | 1.29 (0.37-4.53) | 1.01 (0.27-3.75) | 1.53 (1.20-1.96) | 0.001 |
| Myocardial Infarction (MI) | Ref. | 0.60 (0.21-1.72) | 0.61 (0.21-1.77) | 0.61 (0.21-1.75) | 0.88 (0.53-1.47) | 0.630 |
| All Cause of Mortality | Ref. | 1.09 (0.80-1.48) | 1.08 (0.79-1.47) | 1.09 (0.80-1.49) | 1.04 (0.96-1.13) | 0.349 |

baPWV, brachial-ankle pulse wave velocity.

Per SD, hazard ratio for per standard deviation (78.8 cm/s) change in the bilateral baPWV difference;

Model 1: adjusted age, sex, mean arterial pressure, heart rate, fasting blood glucose, body mass index, low density lipoprotein cholesterol, high density lipoprotein cholesterol, log-transformed high-sensitivity C-reactive protein, uric acid, estimated glomerular filtration rate, smoking status, alcohol intake, physical activity, dominant hand (Left), lipid-lowering drugs, antihypertensive drugs and antihyperglycemic drugs;

Model 2: Model 1+Interarm blood pressure difference, inter-ankle systolic blood pressure difference;

Model 3: Model 2 +baseline brachial-ankle pulse wave velocity.

Table S11. Hazard ratios for bilateral baPWV difference related to cardiovascular disease and all-cause mortality among 36,780 participants after excluding participants with ABI≤0.9.

|  | <80cm/s  (n=27,850) |  | ≥80cm/s (n=8,930) | | |  |  |
| --- | --- | --- | --- | --- | --- | --- | --- |
|  | HR (95%CI) |  |  | HR (95%CI) |  |  |  |
|  |  | Model 1 | Model 2 | Model 3 | Model 4 | Per SD | P value |
| Cardiovascular Disease (CVD) | Ref. | 1.27 (1.10-1.47) | 1.16 (0.99-1.37) | 1.15 (0.97-1.35) | 1.03 (0.87-1.22) | 1.05 (0.99-1.13) | 0.167 |
| Cerebral Infarction (CI) | Ref. | 1.24 (1.05-1.47) | 1.12 (0.92-1.36) | 1.11 (0. 91-1.34) | 1.00 (0.82-1.22) | 1.03 (0.94-1.12) | 0.529 |
| Intracerebral Hemorrhage (CH) | Ref. | 1.28 (0.78-2.09) | 1.19 (0.68-2.08) | 1.19 (0.68-2.08) | 0.99 (0.56-1.78) | 1.19 (1.03-1.37) | 0.017 |
| Myocardial Infarction (MI) | Ref. | 1.35 (0.97-1.89) | 1.28 (0.88-1.87) | 1.24 (0.85-1.82) | 1.15 (0.78-1.71) | 1.01 (0.86-1.20) | 0.868 |
| All Cause of Mortality | Ref. | 1.13 (1.00-1.29) | 1.11 (0.98-1.27) | 1.09 (0.96-1.24) | 1.04 (0. 91-1.18) | 1.07 (1.02-1.12) | 0.006 |

baPWV, brachial-ankle pulse wave velocity.

Per SD, hazard ratio for per standard deviation (78.8 cm/s) change in the bilateral baPWV difference;

Model 1: adjusted age, sex, mean arterial pressure, heart rate, fasting blood glucose, body mass index, low density lipoprotein cholesterol, high density lipoprotein cholesterol, log-transformed high-sensitivity C-reactive protein, uric acid, estimated glomerular filtration rate, smoking status, alcohol intake, physical activity and dominant hand (Left).

Model 2: Model 1 + lipid-lowering drugs, antihypertensive drugs and antihyperglycemic drugs;

Model 3: Model 2+ Interarm blood pressure difference, inter-ankle systolic blood pressure difference;

Model 4: Model 3 + baseline brachial-ankle pulse wave velocity.

Table S12. Hazard ratios for bilateral baPWV difference related to cardiovascular disease and all-cause mortality among 36,353 participants after excluding participants with IAD≥15mmHg.

|  | <80cm/s  (n=27,353) |  | ≥80cm/s (n=9,000) | | |  |  |
| --- | --- | --- | --- | --- | --- | --- | --- |
|  | HR (95%CI) |  |  | HR (95%CI) |  |  |  |
|  |  | Model 1 | Model 2 | Model 3 | Model 4 | Per SD | P value |
| Cardiovascular Disease (CVD) | Ref. | 1.31 (1.13-1.51) | 1.22 (1.04-1.45) | 1.22 (1.03-1.45) | 1.11 (0.94-1.32) | 1.07 (1.02-1.13) | 0.008 |
| Cerebral Infarction (CI) | Ref. | 1.23 (1.03-1.46) | 1.14 (0.93-1.39) | 1.14 (0.93-1.40) | 1.03 (0.84-1.27) | 1.03 (0.96-1.10) | 0.430 |
| Intracerebral Hemorrhage (CH) | Ref. | 1.48 (0.90-2.44) | 1.33 (0.75-2.37) | 1.38 (0.77-2.46) | 1.15 (0.63-2.09) | 1.17 (1.03-1.33) | 0.021 |
| Myocardial Infarction (MI) | Ref. | 1.51 (1.09-2.09) | 1.44 (0.99-2.07) | 1.40 (0.97-2.03) | 1.35 (0.92-1.97) | 1.11 (1.02-1.21) | 0.017 |
| All Cause of Mortality | Ref. | 1.18 (1.04-1.33) | 1.16 (1.02-1.32) | 1.14 (1.01-1.29) | 1.10 (0.96-1.25) | 1.07 (1.04-1.10) | <0.001 |

baPWV, brachial-ankle pulse wave velocity.

Per SD, hazard ratio for per standard deviation (78.8 cm/s) change in the bilateral baPWV difference;

Model 1: adjusted age, sex, mean arterial pressure, heart rate, fasting blood glucose, body mass index, low density lipoprotein cholesterol, high density lipoprotein cholesterol, log-transformed high-sensitivity C-reactive protein, uric acid, estimated glomerular filtration rate, smoking status, alcohol intake, physical activity and dominant hand (Left).

Model 2: Model 1 + lipid-lowering drugs, antihypertensive drugs and antihyperglycemic drugs;

Model 3: Model 2+ Interarm blood pressure difference, inter-ankle systolic blood pressure difference;

Model 4: Model 3 + baseline brachial-ankle pulse wave velocity.

Table S13. Hazard ratios for bilateral baPWV difference related to cardiovascular disease and all-cause mortality among 35,053 participants after excluding participants with ABI≤0.9 or IAD≥15mmHg.

|  | <80cm/s  (n=26,644) |  | | ≥80cm/s (n=8,409) |  | |  |
| --- | --- | --- | --- | --- | --- | --- | --- |
|  | HR (95%CI) |  |  | HR (95%CI) |  |  |  |
|  |  | Model 1 | Model 2 | Model 3 | Model 4 | Per SD | P value |
| Cardiovascular Disease (CVD) | Ref. | 1.25 (1.07-1.45) | 1.15 (0.97-1.37) | 1.14 (0.96-1.36) | 1.03 (0.86-1.24) | 1.05 (0.98-1.13) | 0.188 |
| Cerebral Infarction (CI) | Ref. | 1.20 (1.00-1.43) | 1.09 (0.88-1.33) | 1.08 (0. 88-1.33) | 0.98 (0.79-1.21) | 1.02 (0.93-1.12) | 0.656 |
| Intracerebral Hemorrhage (CH) | Ref. | 1.49 (0.90-2.47) | 1.39 (0.78-2.48) | 1.41 (0.79-2.52) | 1.18 (0.64-2.15) | 1.21 (1.06-1.40) | 0.005 |
| Myocardial Infarction (MI) | Ref. | 1.32 (0.93-1.88) | 1.26 (0.84-1.87) | 1.23 (0.83 -1.84) | 1.13 (0.75 -1.71) | 1.00 (0.84-1.19) | 0.997 |
| All Cause of Mortality | Ref. | 1.11 (0.97-1.26) | 1.10 (0.96-1.25) | 1.07 (0.94-1.23) | 1.02 (0.89-1.17) | 1.07 (1.02-1.12) | 0.007 |

baPWV, brachial-ankle pulse wave velocity.

Per SD, hazard ratio for per standard deviation (78.8 cm/s) change in the bilateral baPWV difference;

Model 1: adjusted age, sex, mean arterial pressure, heart rate, fasting blood glucose, body mass index, low density lipoprotein cholesterol, high density lipoprotein cholesterol, log-transformed high-sensitivity C-reactive protein, uric acid, estimated glomerular filtration rate, smoking status, alcohol intake, physical activity and dominant hand (Left).

Model 2: Model 1 + lipid-lowering drugs, antihypertensive drugs and antihyperglycemic drugs;

Model 3: Model 2+ Interarm blood pressure difference, inter-ankle systolic blood pressure difference;

Model 4: Model 3 + baseline brachial-ankle pulse wave velocity.

Table S14. Time-dependent analysis for bilateral baPWV difference related to cardiovascular disease and all-cause mortality among 38,356 participants.

|  | <80cm/s (n=28,705) |  | >80cm/s (n=9,651) | | |  |  |
| --- | --- | --- | --- | --- | --- | --- | --- |
|  | HR (95%CI) |  | HR (95%CI) | | |  |  |
|  |  | Model 1 | Model 2 | Model 3 | Model 4 | Per SD | P value |
| Cardiovascular disease | Ref. | 1.21 (1.08-1.37) | 1.18 (1.04-1.33) | 1.18 (1.04-1.33) | 1.12 (0.99-1.28) | 1.03 (0.99-1.07) | 0.126 |
| Cerebral infarction | Ref. | 1.19 (1.04-1.07) | 1.15 (1.00-1.33) | 1.16 (1.00-1.34) | 1.10 (0.94-1.27) | 1.02 (0.98-1.07) | 0.375 |
| Intracerebral hemorrhage | Ref. | 1.16 (0.77-1.75) | 1.22 (0.81-1.84) | 1.23 (0.81-1.86) | 1.14 (0.74-1.74) | 1.18 (1.00-1.26) | 0.062 |
| Myocardial infarction | Ref. | 1.33 (1.00-1.77) | 1.25 (0.94-1.67) | 1.23 (0.92-1.64) | 1.24 (0.92-1.67) | 1.02 (1.94-1.12) | 0.573 |
| All-cause mortality | Ref. | 1.11 (0.98-1.27) | 1.09 (0.96-1.25) | 1.08 (0.94-1.23) | 1.07 (0.94-1.23) | 1.06 (1.03-1.09) | <0.001 |

baPWV, brachial-ankle pulse wave velocity.

Per SD, hazard ratio for per standard deviation (78.8 cm/s) change in the bilateral baPWV difference;

Model 1: adjusted age, sex, mean arterial pressure, heart rate, fasting blood glucose, body mass index, low density lipoprotein cholesterol, high density lipoprotein cholesterol, log-transformed high-sensitivity C-reactive protein, uric acid, estimated glomerular filtration rate, smoking status, alcohol intake, physical activity and dominant hand (Left).

Model 2: Model 1 + lipid-lowering drugs, antihypertensive drugs and antihyperglycemic drugs;

Model 3: Model 2+Interarm blood pressure difference, inter-ankle systolic blood pressure difference;

Model 4: Model 3 +baseline brachial-ankle pulse wave velocity.

Table S15. Hazard ratios (95% confidence interval) of cardiovascular disease and all-cause mortality in groups stratified by the bilateral difference in the brachial-ankle pulse wave velocity (BPWVD) and blood pressure status (n=38,356).

|  |  | Normotension with low BPWVD (n=17,587) | Hypertension with low BPWVD (n=1,1118) | Normotension with high BPWVD (n=4,284) | Hypertension with high BPWVD (n=5,367) |
| --- | --- | --- | --- | --- | --- |
| Cardiovascular disease | Model 1 | Ref. | 2.70 (2.18-3.34) | 1.55 (1.14-2.12) | 3.12 (2.47-3.93) |
|  | Model 2 | Ref. | 2.65 (2.14-3.28) | 1.54 (1.13-2.10) | 3.04 (2.41-3.83) |
|  | Model 3 | Ref. | 2.37 (1.91-2.95) | 1.42 (1.04-1.94) | 2.47 (1.93-3.15) |
| Cerebral infarction | Model 1 | Ref. | 2.92 (2.26-3.79) | 1.42 (0.96-2.11) | 3.09 (2.33-4.10) |
|  | Model 2 | Ref. | 2.87 (2.21-3.72) | 1.41 (0.95-2.09) | 3.00 (2.26-3.99) |
|  | Model 3 | Ref. | 2.49 (1.91-3.24) | 1.26 (0.85-1.87) | 2.30 (1.70-3.10) |
| Intracerebral hemorrhage | Model 1 | Ref. | 3.63 (1.67-7.89) | 2.77 (1.03-7.48) | 5.04 (2.20-11.57) |
|  | Model 2 | Ref. | 3.59 (1.65-7.81) | 2.76 (1.02-7.46) | 4.96 (2.16-11.41) |
|  | Model 3 | Ref. | 3.22 (1.46-7.08) | 2.55 (0.94-6.94) | 4.01 (1.67-9.58) |
| Myocardial infarction | Model 1 | Ref. | 1.89 (1.22-2.93) | 1.56 (0.85-2.87) | 2.67 (1.68-4.25) |
|  | Model 2 | Ref. | 1.85 (1.19-2.88) | 1.55 (0.85-2.85) | 2.61 (1.63-4.16) |
|  | Model 3 | Ref. | 1.83 (1.17-2.87) | 1.54 (0.84-2.83) | 2.56 (1.56-4.18) |
| All-cause mortality | Model 1 | Ref. | 1.08 (0.90-1.30) | 1.28 (1.01-1.62) | 1.33 (1.10-1.61) |
|  | Model 2 | Ref. | 1.07 (0.89-1.29) | 1.27 (1.00-1.60) | 1.30 (1.07-1.58) |
|  | Model 3 | Ref. | 1.01 (0.84-1.23) | 1.21 (0.96-1.54) | 1.18 (0.96-1.45) |

Hypertension was defined as systolic/diastolic blood pressure >140/90 mmHg, or using antihypertensive drugs;

Model 1: adjusted for age, sex, mean arterial pressure, heart rate, fasting blood glucose, body mass index, low density lipoprotein cholesterol, high density lipoprotein cholesterol, log-transformed high-sensitivity C-reactive protein, uric acid, estimated glomerular filtration rate, smoking status, alcohol intake, physical activity and dominant hand, blood lipid-lowering drugs, and antihyperglycemic drugs;

Model 2: Model 1 + Interarm blood pressure difference, inter-ankle systolic blood pressure difference;

Model 3: Model 2 + baseline brachial ankle pulse wave velocity

Table S16. Hazard ratios of cardiovascular disease and all-cause mortality in groups stratified by the bilateral difference in the brachial-ankle pulse wave velocity (BPWVD) and different fasting blood glucose concentration status (n=38,356).

|  |  | Non-diabetes with low BPWVD (n=25,205) | Diabetes with low BPWVD (n=3,500) | Non-diabetes with high BPWVD (n=7,488) | Diabetes with high BPWVD (n=2,163) |
| --- | --- | --- | --- | --- | --- |
| Cardiovascular disease | Model 1 | Ref. | 1.98 (1.60-2.43) | 1.35 (1.13-1.61) | 2.22 (1.78-2.78) |
|  | Model 2 | Ref. | 1.98 (1.61-2.44) | 1.33 (1.12-1.59) | 2.21 (1.77-2.76) |
|  | Model 3 | Ref. | 1.85 (1.50-2.29) | 1.17 (0.98-1.40) | 1.88 (1.50-2.36) |
| Cerebral infarction | Model 1 | Ref. | 1.96 (1.53-2.51) | 1.18 (0.95-1.46) | 2.25 (1.73-2.92) |
|  | Model 2 | Ref. | 1.97 (1.54-2.53) | 1.16 (0.93-1.44) | 2.24 (1.73-2.91) |
|  | Model 3 | Ref. | 1.81 (1.41-2.32) | 0.98 (0.78-1.23) | 1.83 (1.40-2.39) |
| Intracerebral hemorrhage | Model 1 | Ref. | 1.32 (0.59-2.95) | 1.88 (1.09-3.23) | 1.27 (0.51-3.18) |
|  | Model 2 | Ref. | 1.31 (0.59-2.93) | 1.86 (1.08-3.21) | 1.25 (0.50-3.14) |
|  | Model 3 | Ref. | 1.24 (0.55-2.77) | 1.65 (0.94-2.90) | 1.05 (0.41-2.68) |
| Myocardial infarction | Model 1 | Ref. | 2.27 (1.45-3.58) | 1.72 (1.19-2.49) | 2.47 (1.52-4.01) |
|  | Model 2 | Ref. | 2.26 (1.44-3.56) | 1.71 (1.18-2.46) | 2.44 (1.50-3.96) |
|  | Model 3 | Ref. | 2.23 (1.41-3.51) | 1.66 (1.14-2.42) | 2.36 (1.44-3.86) |
| All-cause mortality | Model 1 | Ref. | 1.64 (1.33-2.03) | 1.29 (1.11-1.52) | 1.96 (1.60-2.40) |
|  | Model 2 | Ref. | 1.65 (1.34-2.04) | 1.27 (1.09-1.49) | 1.94 (1.58-2.38) |
|  | Model 3 | Ref. | 1.60 (1.30-1.98) | 1.21 (1.03-1.42) | 1.83 (1.48-2.25) |

Diabetes was defined as either FBG ≥7.0 mmol/L, self-report of a physician diagnosis, or self-report use of antidiabetic medication.

Per SD, hazard ratio for per standard deviation (78.8 cm/s) change in the bilateral baPWV difference;

Model 1: adjusted for age, sex, mean arterial pressure, heart rate, body mass index, low density lipoprotein cholesterol, high density lipoprotein cholesterol, log-transformed high-sensitivity C-reactive protein, uric acid, estimated glomerular filtration rate, smoking status, alcohol intake, physical activity and dominant hand, blood lipid-lowering drugs, and antihypertensive drugs;

Model 2: Model 1 + Interarm blood pressure difference, inter-ankle systolic blood pressure difference;

Model 3: Model 2 + baseline baPWV.

Table S17. Hazard ratios of cardiovascular disease and all-cause mortality in groups stratified by the bilateral difference in the brachial-ankle pulse wave velocity (BPWVD) and overweight status (n=38,356)

|  |  | BMI<24 kg/m^2^ with low BPWVD (n=11,278) | BMI≥24 kg/m^2^ with low BPWVD (n=17,427) | BMI<24 kg/m^2^ with high BPWVD (n=3,273) | BMI≥24 kg/m^2^ with high BPWVD (n=6,378) |
| --- | --- | --- | --- | --- | --- |
| Cardiovascular disease | Model 1 | Ref. | 1.40 (1.14-1.71) | 1.33 (1.02-1.75) | 1.84 (1.48-2.29) |
|  | Model 2 | Ref. | 1.38 (1.13-1.69) | 1.32 (1.00-1.73) | 1.80 (1.44-2.24) |
|  | Model 3 | Ref. | 1.40 (1.15-1.72) | 1.13 (0.86-1.49) | 1.61 (1.29-2.00) |
| Cerebral infarction | Model 1 | Ref. | 1.45 (1.14-1.84) | 1.17 (0.84-1.63) | 1.78 (1.37-2.31) |
|  | Model 2 | Ref. | 1.43 (1.12-1.82) | 1.15 (0.83-1.61) | 1.74 (1.33-2.26) |
|  | Model 3 | Ref. | 1.45 (1.14-1.85) | 0.95 (0.68-1.34) | 1.51 (1.16-1.97) |
| Intracerebral hemorrhage | Model 1 | Ref. | 1.12 (0.57-2.21) | 1.23 (0.48-3.13) | 2.05 (1.01-4.15) |
|  | Model 2 | Ref. | 1.11 (0.56-2.19) | 1.21 (0.48-3.10) | 2.01 (0.99-4.09) |
|  | Model 3 | Ref. | 1.11 (0.56-2.20) | 1.06 (0.41-2.73) | 1.81 (0.89-3.71) |
| Myocardial infarction | Model 1 | Ref. | 1.37 (0.87-2.13) | 1.91 (1.11-3.28) | 1.91 (1.19-3.08) |
|  | Model 2 | Ref. | 1.35 (0.86-2.11) | 1.89 (1.10-3.24) | 1.88 (1.17-3.02) |
|  | Model 3 | Ref. | 1.36 (0.87-2.12) | 1.82 (1.05-3.14) | 1.82 (1.13-2.94) |
| All-cause mortality | Model 1 | Ref. | 0.82 (0.69-0.99) | 1.13 (0.92-1.39) | 1.17 (0.97-1.41) |
|  | Model 2 | Ref. | 0.83 (0.69-0.99) | 1.11 (0.90-1.37) | 1.16 (0.96-1.39) |
|  | Model 3 | Ref. | 0.83 (0.69-1.00) | 1.05 (0.85-1.29) | 1.11 (0.92-1.33) |

BMI, body mass index;

Per SD, hazard ratio for per standard deviation (78.8 cm/s) change in the bilateral baPWV difference;

Model 1: adjusted for age, sex, mean arterial pressure, heart rate, fasting blood glucose, low density lipoprotein cholesterol, high density lipoprotein cholesterol, log-transformed high-sensitivity C-reactive protein, uric acid, estimated glomerular filtration rate, smoking status, alcohol intake, physical activity and dominant hand, blood lipid-lowering drugs, antihypertensive drugs and antihyperglycemic drugs;

Model 2: Model 1 + Interarm blood pressure difference, inter-ankle systolic blood pressure difference;

Model 3: Model 2 + baseline baPWV.

Table S18. Hazard ratios of cardiovascular disease and all-cause mortality in groups stratified by the bilateral difference in the brachial-ankle pulse wave velocity (BPWVD) and obesity status (n=38,356)

|  |  | BMI<28 kg/m^2^ with low BPWVD (n=24,198) | BMI≥28 kg/m^2^ with low BPWVD (n=4,507) | BMI<28 kg/m^2^ with high BPWVD (n=7,780) | BMI≥28 kg/m^2^ with high BPWVD (n=1,871) |
| --- | --- | --- | --- | --- | --- |
| Cardiovascular disease | Model 1 | Ref. | 1.55 (1.25-1.91) | 1.48 (1.26-1.75) | 1.35 (1.02-1.78) |
|  | Model 2 | Ref. | 1.52 (1.23-1.88) | 1.46 (1.24-1.72) | 1.32 (1.00-1.74) |
|  | Model 3 | Ref. | 1.56 (1.26-1.92) | 1.28 (1.08-1.51) | 1.19 (0.90-1.57) |
| Cerebral infarction | Model 1 | Ref. | 1.55 (1.20-1.99) | 1.39 (1.14-1.69) | 1.14 (0.80-1.62) |
|  | Model 2 | Ref. | 1.52 (1.18-1.95) | 1.37 (1.12-1.66) | 1.12 (0.79-1.59) |
|  | Model 3 | Ref. | 1.56 (1.21-2.01) | 1.16 (0.94-1.41) | 0.98 (0.69-1.40) |
| Intracerebral hemorrhage | Model 1 | Ref. | 2.11 (1.05-4.21) | 1.68 (0.93-3.02) | 3.19 (1.51-6.75) |
|  | Model 2 | Ref. | 2.10 (1.05-4.20) | 1.67 (0.92-3.01) | 3.14 (1.48-6.66) |
|  | Model 3 | Ref. | 2.13 (1.07-4.26) | 1.47 (0.80-2.69) | 2.86 (1.34-6.09) |
| Myocardial infarction | Model 1 | Ref. | 1.34 (0.83-2.17) | 1.69 (1.20-2.38) | 1.44 (0.80-2.58) |
|  | Model 2 | Ref. | 1.33 (0.82-2.15) | 1.68 (1.19-2.36) | 1.41 (0.79-2.53) |
|  | Model 3 | Ref. | 1.33 (0.82-2.16) | 1.62 (1.14-2.30) | 1.37 (0.76-2.47) |
| All-cause mortality | Model 1 | Ref. | 1.02 (0.80-1.30) | 1.27 (1.10-1.47) | 1.43 (1.11-1.84) |
|  | Model 2 | Ref. | 1.02 (0.80-1.30) | 1.25 (1.08-1.45) | 1.43 (1.11-1.84) |
|  | Model 3 | Ref. | 1.03 (0.81-1.31) | 1.18 (1.02-1.38) | 1.37 (1.06-1.76) |

BMI, body mass index;

Per SD, hazard ratio for per standard deviation (78.8 cm/s) change in the bilateral baPWV difference;

Model 1: adjusted for age, sex, mean arterial pressure, heart rate, fasting blood glucose, low density lipoprotein cholesterol, high density lipoprotein cholesterol, log-transformed high-sensitivity C-reactive protein, uric acid, estimated glomerular filtration rate, smoking status, alcohol intake, physical activity and dominant hand, blood lipid-lowering drugs, antihypertensive drugs and antihyperglycemic drugs;

Model 2: Model 1 + Interarm blood pressure difference, inter-ankle systolic blood pressure difference;

Model 3: Model 2 + baseline baPWV.

Table S19. Hazard ratios for bilateral baPWV difference related to cardiovascular disease and all-cause mortality after performing propensity score matching with consideration of several important factors including age, sex, systolic blood pressure, and heart rate.

|  | <80cm/s (n=9,376) |  | >80cm/s (n=9,376) | | |  |
| --- | --- | --- | --- | --- | --- | --- |
|  | HR (95%CI) |  | HR (95%CI) | | |  |
|  |  | Model 1 | Model 2 | Model 3 | Per SD | P value |
| Cardiovascular disease | Ref. | 1.34 (1.17-1.54) | 1.29 (1.13-1.48) | 1.13 (0.99-1.31) | 1.07 (1.03-1.12) | 0.002 |
| Cerebral infarction | Ref. | 1.25 (1.07-1.47) | 1.20 (1.02-1.40) | 1.06 (0.90-1.24) | 1.06 (1.01-1.11) | 0.015 |
| Intracerebral hemorrhage | Ref. | 1.22 (0.77-1.93) | 1.27 (0.79-2.02) | 1.05 (0.65-1.69) | 1.13 (1.00-1.28) | 0.058 |
| Myocardial infarction | Ref. | 1.89 (1.34-2.67) | 1.70 (1.20-2.41) | 1.59 (1.12-2.26) | 1.09 (1.00-1.18) | 0.048 |
| All-cause mortality | Ref. | 1.38 (1.21-1.57) | 1.28 (1.12-1.46) | 1.07 (0.93-1.23) | 1.10 (1.07-1.13) | <0.001 |

baPWV, brachial-ankle pulse wave velocity.

Per SD, hazard ratio for per standard deviation (78.8 cm/s) change in the bilateral baPWV difference;

Model 1: crude model;

Model 2: adjusted Interarm blood pressure difference, inter-ankle systolic blood pressure difference, fasting blood glucose, body mass index, low density lipoprotein cholesterol, high density lipoprotein cholesterol, log-transformed high-sensitivity C-reactive protein, uric acid, estimated glomerular filtration rate, smoking status, alcohol intake, physical activity, dominant hand (Left), and anti-lipotropic drugs, antihypertensive drugs and antihyperglycemic drugs.

Model 3: Model 2 +baseline brachial-ankle pulse wave velocity.

Table S20. Time-dependent area under the receiver operating characteristic curve for cardiovascular diseases and all-cause mortality by BPWVD, ABI and IAD.

|  | Follow-up years | | | | |
| --- | --- | --- | --- | --- | --- |
|  | T=2 | T=4 | T=6 | T=8 | T=10 |
| BPWVD |  |  |  |  |  |
| Cardiovascular diseases | 62.1% | 62.9% | 61.1% | 59.4% | 72.7% |
| All-cause of Mortality | 65.9% | 65.7% | 65.0% | 64.2% | 76.2% |
| ABI |  |  |  |  |  |
| Cardiovascular diseases | 50.5% | 49.9% | 49.9% | 50.0% | 50.0% |
| All-cause of Mortality | 49.8% | 49.3% | 49.4% | 49.5% | 49.5% |
| IAD |  |  |  |  |  |
| Cardiovascular diseases | 45.0% | 43.1% | 44.5% | 46.8% | 35.8% |
| All-cause of Mortality | 47.5% | 46.9% | 48.2% | 50.9% | 39.7% |

ABI, ankle-brachial index, IAD, Interarm blood pressure difference, BPWVD, bilateral brachial-ankle Pulse Wave Velocity Difference.


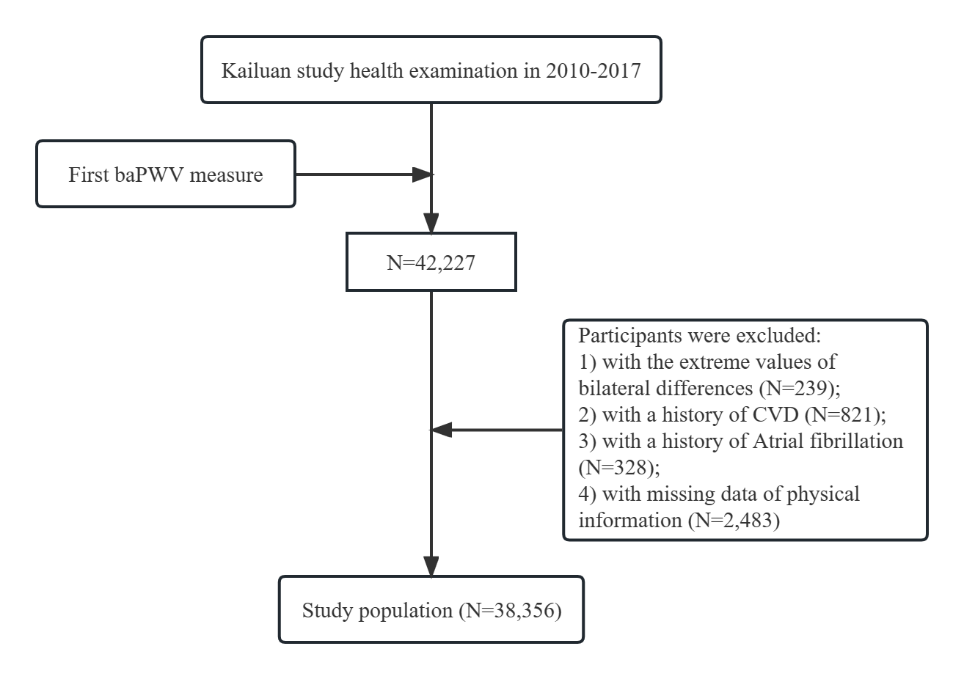


Figure S1. Follow chart.


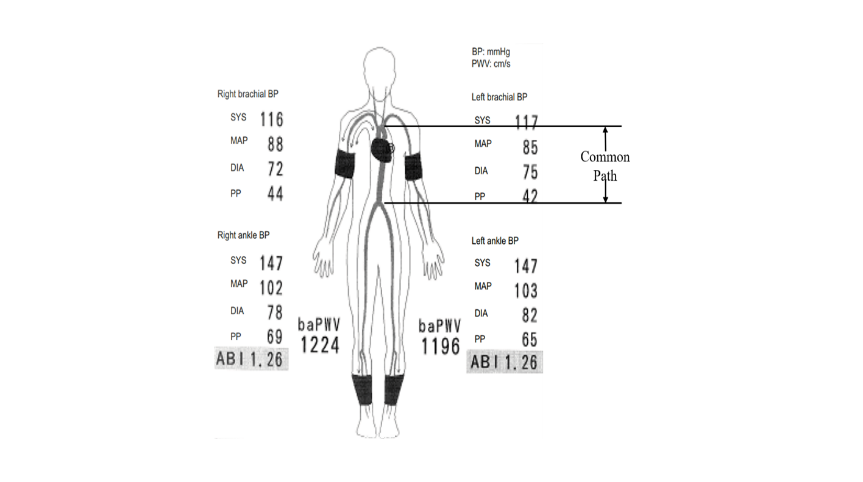


Figure S2. System for measuring baPWV.


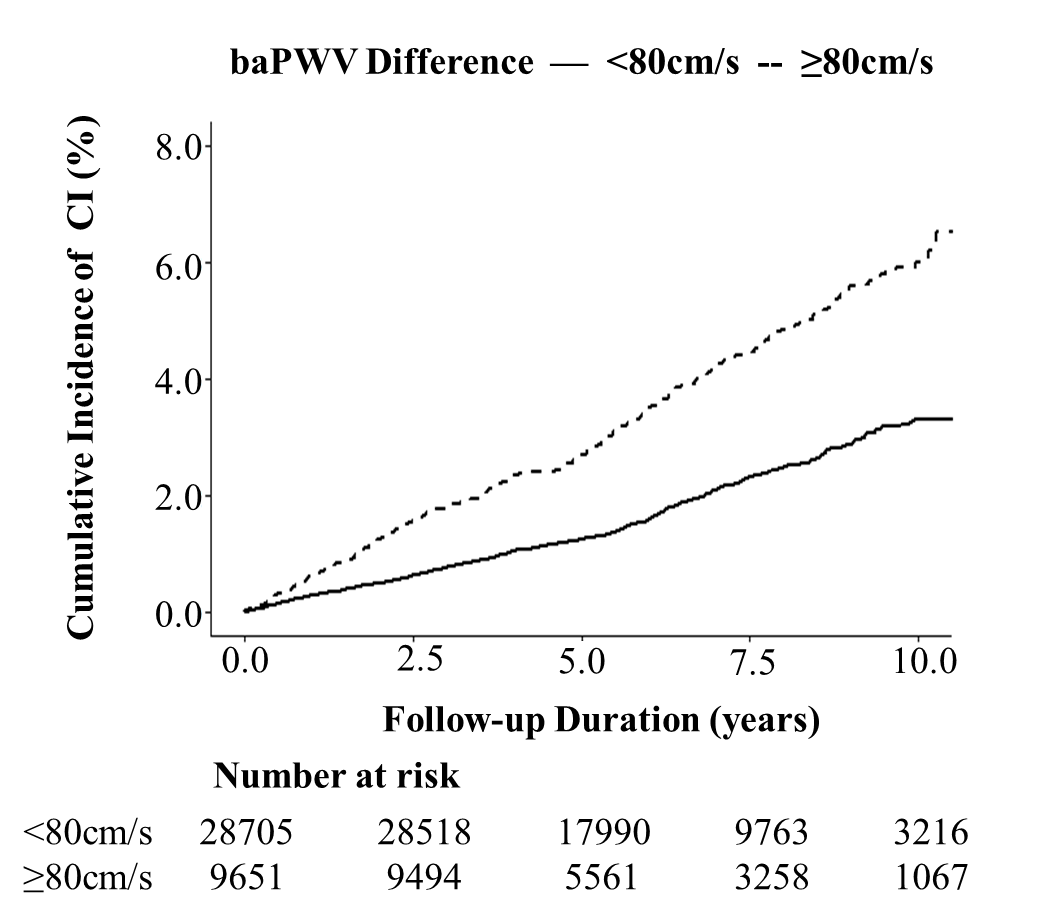


Figure S3. Kaplan-Meier plot of the cumulative incidence of Cerebral Infarction over a mean of 6.19 years among 38,356 participants with high or normal bilateral baPWV difference.


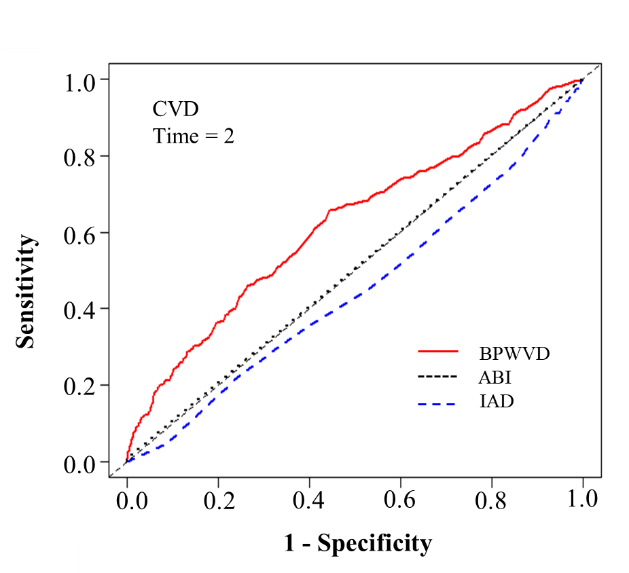

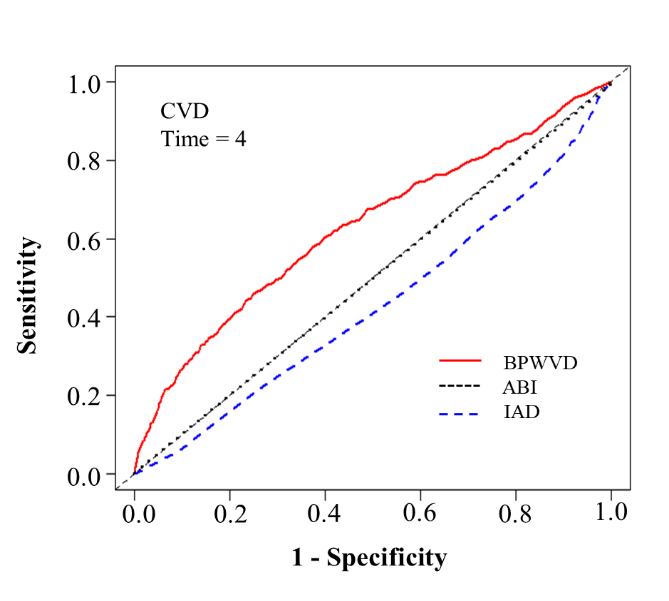

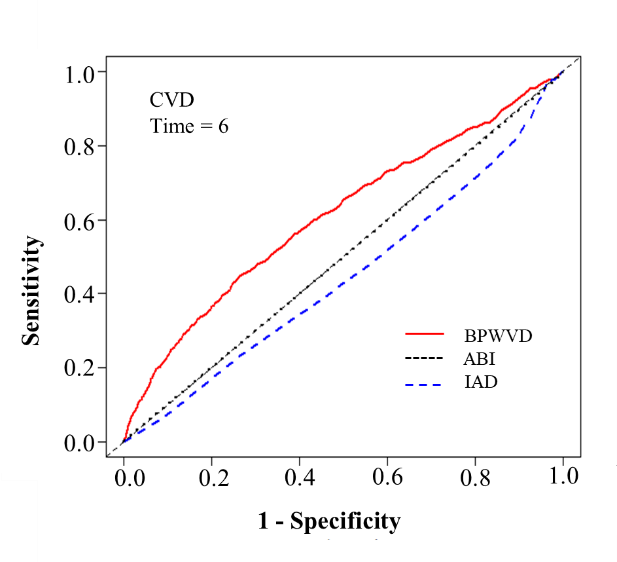


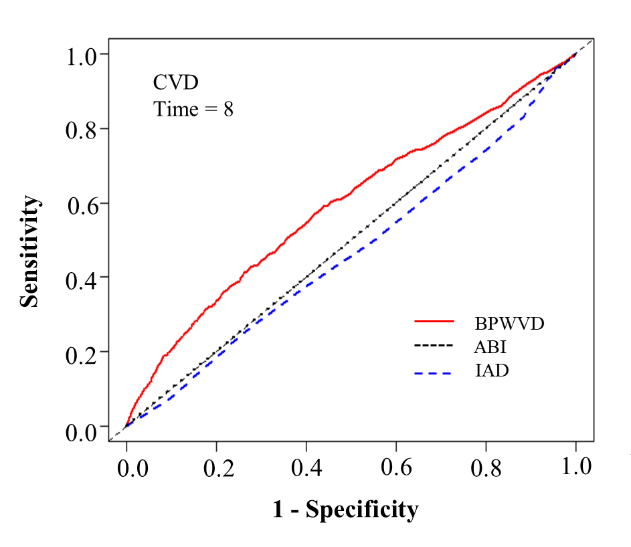

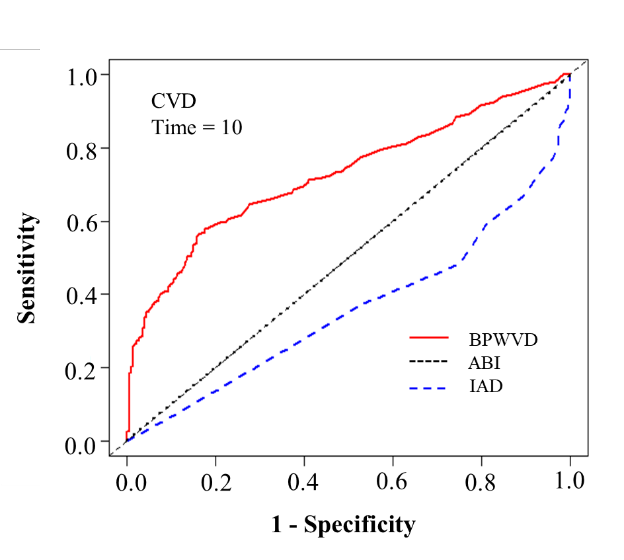


Figure S4. ROC curves for cardiovascular diseases by BPWVD, ABI and IAD at 2, 4, 6, 8 and 10 years by using the Aalen method. The diagonal line in each plot is included for reference.

ABI, ankle-brachial index, IAD, Interarm blood pressure difference, BPWVD, bilateral brachial-ankle Pulse Wave Velocity Difference.


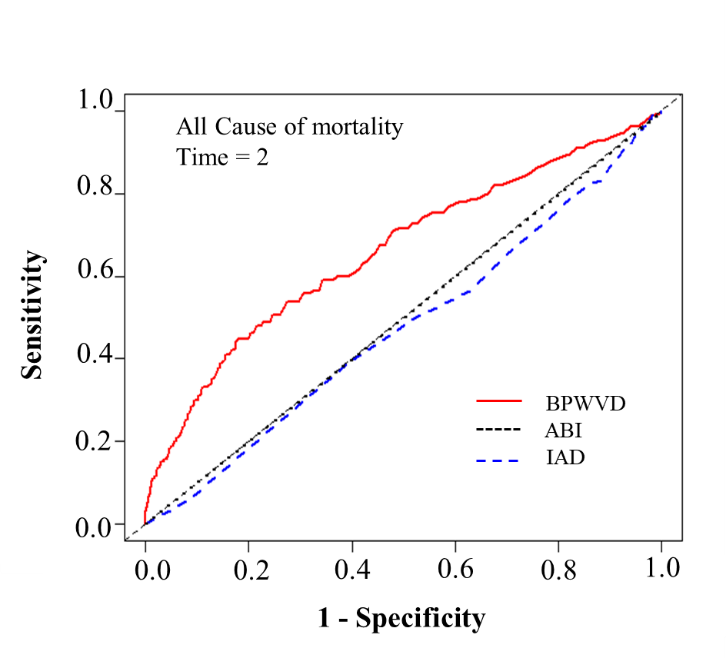

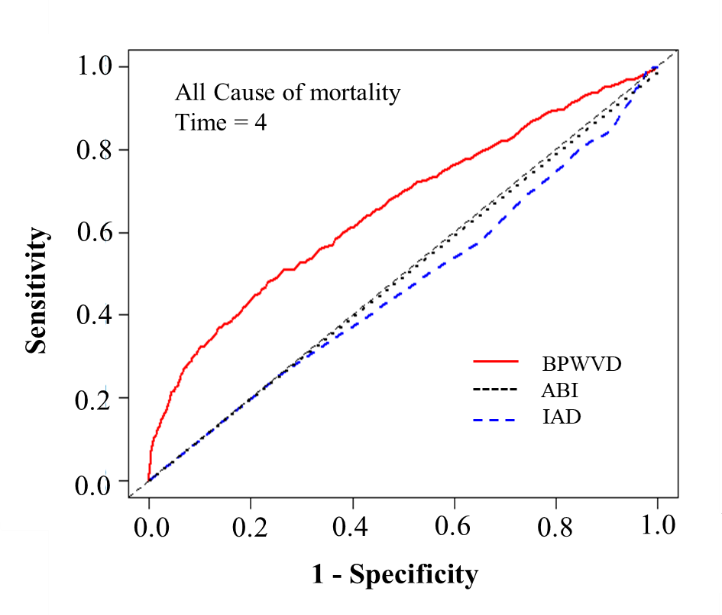

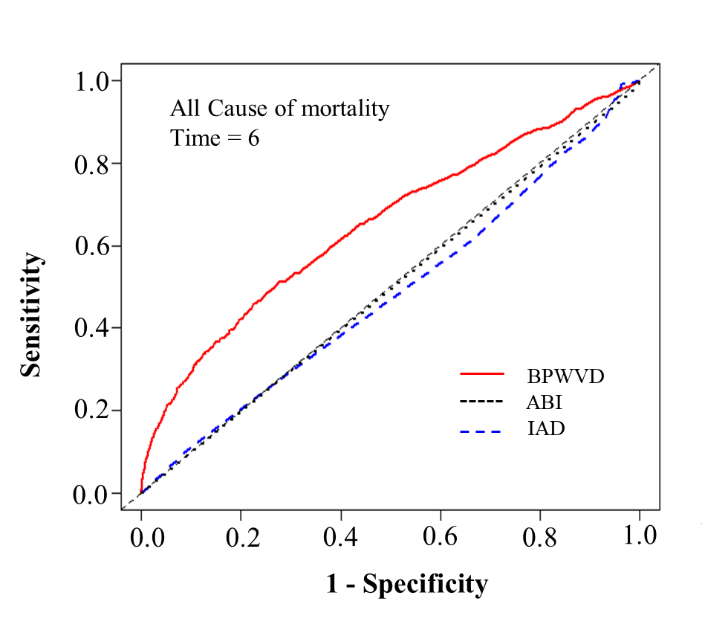


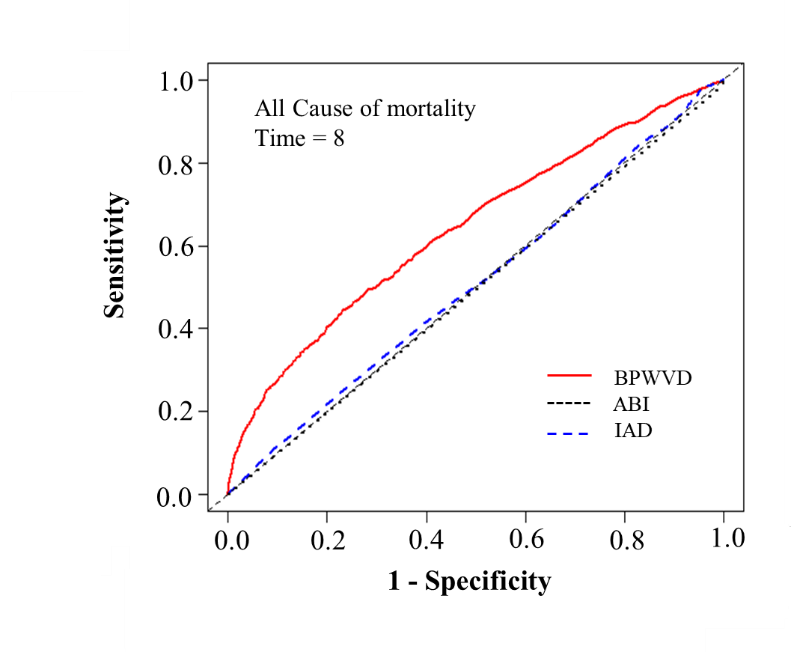

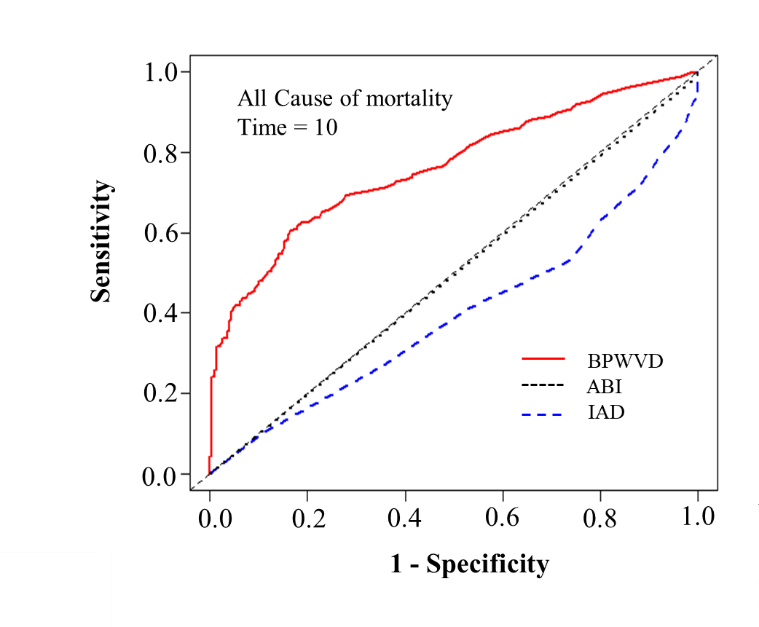


Figure S5. ROC curves for all-cause mortality by BPWVD, ABI and IAD at 2, 4, 6, 8 and 10 years by using the Aalen method. The diagonal line in each plot is included for reference.

ABI, ankle-brachial index, IAD, Interarm blood pressure difference, BPWVD, bilateral brachial-ankle Pulse Wave Velocity Difference.
